# Epitope-Directed Antibody Elicitation by Genetically Encoded Chemical Cross-Linking Reactivity in the Antigen

**DOI:** 10.1021/acscentsci.3c00265

**Published:** 2023-06-06

**Authors:** Chaoyang Zhu, Liang Xu, Longxin Chen, Zihan Zhang, Yuhan Zhang, Weiping Wu, Chengxiang Li, Shuang Liu, Shuqin Xiang, Shengwang Dai, Jay Zhang, Hui Guo, Yinjian Zhou, Feng Wang

**Affiliations:** † Key Laboratory of Protein and Peptide Pharmaceutical, Institute of Biophysics, Chinese Academy of Sciences, Beijing 100101, China; ‡ College of Life Sciences, University of Chinese Academy of Sciences, Beijing 100101, China; § Molecular Biology Laboratory, Zhengzhou Normal University, Zhengzhou 450044, China; ∥ Suzhou Institute for Biomedical Research, Suzhou, Jiangsu 215028, China; ⊥ Beijing Translational Center for Biopharmaceuticals, Beijing 100101, China

## Abstract

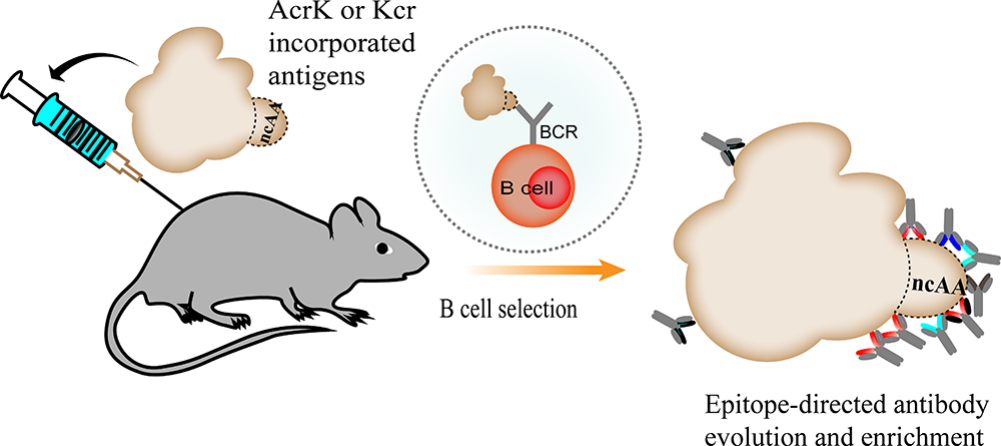

No current methods can selectively elicit an antibody response to a specific conformational epitope in a whole antigen in vivo. Here, we incorporated *N*ε-acryloyl-l-lysine (AcrK) or *N*ε-crotonyl-l-lysine (Kcr) with cross-linking activities into the specific epitopes of antigens and immunized mice to generate antibodies that can covalently cross-link with the antigens. By taking advantage of antibody clonal selection and evolution in vivo, an orthogonal antibody–antigen cross-linking reaction can be generated. With this mechanism, we developed a new approach for facile elicitation of antibodies binding to specific epitopes of the antigen in vivo. Antibody responses were directed and enriched to the target epitopes on protein antigens or peptide-KLH conjugates after mouse immunization with the AcrK or Kcr-incorporated immunogens. The effect is so prominent that the majority of selected hits bind to the target epitope. Furthermore, the epitope-specific antibodies effectively block IL-1β from activating its receptor, indicating its potential for the development of protein subunit vaccines.

## Abstract 2

A new approach was developed for elicitation of epitope-directed antibody response in vivo. Antibody responses were directed and enriched to the target epitopes after mouse immunization with the AcrK or Kcr-incorporated immunogens.

## Introduction

The antibody is an important part of the adaptive immunity of vertebrates, playing essential roles in preventing bacterial and viral infections and neutralizing most foreign harmful substances.^1^ In addition, monoclonal antibodies have the advantages of strong affinity, high specificity, good biocompatibility, and Fc-mediated cellular effects, making them a significant class of therapeutics in the treatment of many diseases.^2−4^ Mouse hybridoma technology is one of the most popular methods to generate monoclonal antibodies with high affinities.^5,6^ In recent years, as transgenic mice with human antibody gene fragments have been developed to address the antibody humanization issue, mouse immunization coupled with downstream monoclonal antibody selection has become a major approach to identifying and developing therapeutic antibodies.^7−9^ However, this approach does not always generate functional antibodies effectively.^10^ Therapeutic antibodies must bind to a specific epitope of the target to exert desired functions, such as blocking ligand–receptor interactions as an antagonist or inducing receptor-mediated downstream signaling as an agonist.^11^ Unfortunately, the effective epitope may account only for a small surface area of the whole target protein.^12^ By current methods, if the entire antigen protein is used as the immunogen to immunize mice, the probability of eliciting an antibody response to the desired epitope might be slim. In addition, nonfunctional immune dominant B-cell epitopes on the target could further reduce this probability. One has to evaluate numerous single clones often through high-throughput screening to hopefully identify the functional hits.^13^ To make it even worse, for human targets that have high sequence and structural similarity to mouse homologues, it may become extremely difficult to elicit an antibody response to the functional epitope due to the host’s immune tolerance.^14−16^ This situation often applies to many important drug targets, for example, GPCRs. To overcome this challenge, a few approaches have been developed by modification or grafting of target epitopes to increase the odds. For instance, widespread neutralizing antibodies can be induced by modifying the glycosylation epitope of HIV envelope proteins.^17^

Through screening and selection, engineered tRNA/aaRS orthogonal pairs can be obtained to specifically recognize noncanonical amino acids (ncAAs) and incorporate them at the encoded site of proteins in living host cells.^18^ Since ncAAs have a variety of chemical groups in the side chains to endow proteins with new functions, they have enabled unique archives in studies of protein structure and function, cell imaging, therapeutic protein conjugation, etc.^19^ Previously, we had taken advantage of a photo-cross-linking ncAA, *p*-benzoyl-l-phenylalanine (pBpa), with pBpa incorporated antigens to successfully select antibodies to the target epitope by photo-cross-linking panning from antibody phage display libraries.^20^ This represents the first method with an epitope-specific modality that potentially can be applied to all types of antigens. However, this method relies on in vitro panning phage libraries to select existing hits in the library. *p*-Nitrophenylalanine (pNO_2_F), another ncAA, is a derivative of phenylalanine. It was previously shown that mouse TNF-α and C5a incorporated with pNO_2_F were able to induce an antibody response to broadly target the antigen. However, epitope specificity of the elicited antibodies was not observed, and the authors concluded that pNO_2_F serves as an adjuvant to break T cell tolerance of the host and secondarily enhances the humoral immune response.^21−24^

**Figure 1:**
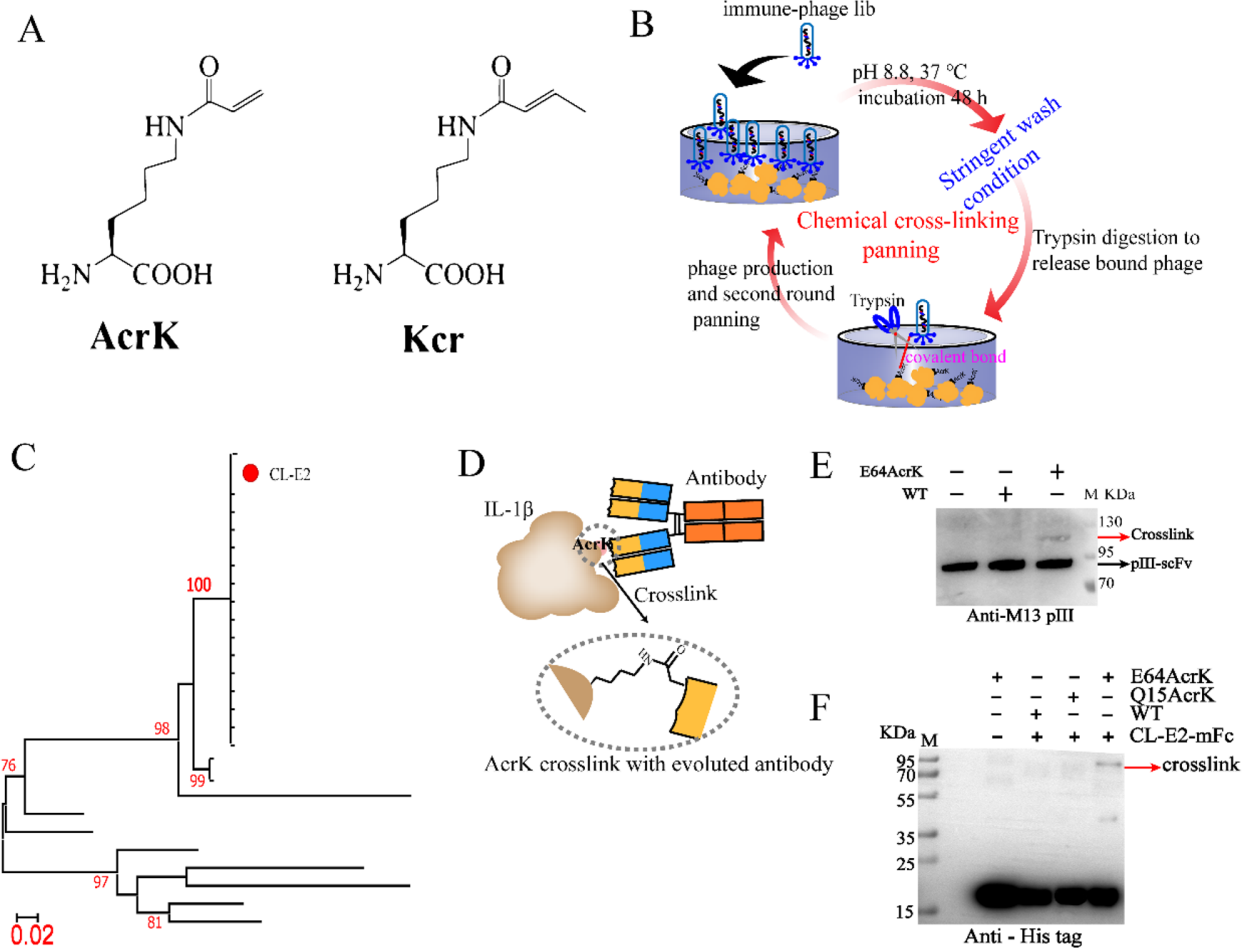
AcrK incorporated hIL-1β elicits antibodies with cross-linking activity. (A) Chemical structures of AcrK and Kcr. (B) Chemical cross-linking panning procedure. (C) Sequence analysis of the hits from chemical cross-linking panning. Among the randomly picked 28 hits, 17 hits showed identical scFv amino acid sequences. The red solid circle represents the clone selected for antigen-binding epitope verification. (D) The cartoon of IL-1βE64AcrK chemical cross-linking with antibodies mediated by AcrK. (E) CL-E2 phage cross-linked with E64AcrK. 10^8^ pfu phages were incubated with E64AcrK at 37 °C for 48 h. The pIII-scFv and the cross-linked complex were detected by Western blot using an anti-pIII antibody. The band corresponding to the cross-linked complex is indicated with a red arrow. (F) CL-E2-mFc protein cross-linked with E64AcrK. WT hIL-1β, Q15AcrK, or E64AcrK were incubated with CL-E2-mFc in a 2:1 molar ratio at pH 8.8 and 37 °C for 48 h. The band corresponding to the cross-linked complex is indicated with a red arrow.

Kcr and AcrK (Figure 1A) are lysine analogs, which can be site-specifically incorporated into proteins by the BuKRS-tRNA^CUA^ and PrKRS-tRNA^CUA^ pairs, respectively.^25,26^ Both AcrK and Kcr have an electron-deficient olefin, which has the potential to form a covalent bond with a lysine or cystine in its proximity. We hypothesized that immunizing mice with AcrK or Kcr incorporated antigens would elicit and enrich antibody responses to the incorporated epitopes due to the chemical cross-linking reactivity of AcrK or Kcr. Here, we incorporated AcrK or Kcr on different epitopes of human interleukin-1β (hIL-1β) and nucleoside triphosphate transporter 2 from *Phaeodactylum tricornutum* (*Pt*NTT2) peptide and demonstrated that antigens incorporated with single chemically reactive amino acids can effectively elicit epitope-directed antibody responses in mice.

## Results

### AcrK Incorporated hIL-1β Elicits Antibodies with Orthogonal Cross-Linking Activity in Vivo

In the process of humoral immunity, the production and maturation of antibodies are highly dependent on the affinity between antigens and B-cell receptors (BCRs).^27,28^ Immune dominant epitopes on antigens with strong affinity can induce the proliferation and differentiation of the corresponding B cells, thus secreting more antibodies binding to those corresponding epitopes. Therefore, the artificial design of immune-dominant epitopes may benefit epitope-oriented antibody evolution. We reason that when mice are immunized with an AcrK incorporated antigen, during the humoral immunity process, a nucleophile residue, such as lysine or cysteine, with suitable distance and geometry could potentially be evolved in the context of BCR to enable cross-linking with the AcrK incorporated antigen. Since a covalently bound antigen-BCR has infinite affinity, AcrK incorporated epitopes become “super” dominant epitopes in antibody elicitation and evolution.

We selected human IL-1β for an initial test of this hypothesis. IL-1β is a pro-inflammatory cytokine that binds to interleukin-1 receptor I (IL1RI) and interleukin-1 receptor II (IL1RII).^29^ Blocking the binding of IL-1β to IL1RI can potentially treat a variety of inflammatory diseases.^30^ Canakinumab is a high affinity antibody that binds to IL-1β with partial paratope overlapped with IL1RI. Based on the structure of the hIL-1β-Canakinumab Fab complex (PDB: 4G6J),^31^ the residue E64, which is the key binding site of hIL-1β to canakinumab (Figure S1), was selected for AcrK incorporation. The PrKRS-tRNA^CUA^ orthogonal pair (encoding AcrK) was inserted in a pEvol vector and cotransformed along with the pET28a-hIL-1βE64TAG plasmid into *E. coli* BL21(DE3). Through amber suppression, the mutant protein E64AcrK was expressed and purified through Ni-NTA chromatography followed by size exclusion chromatography (SEC; Figure S2A). The purity and integrity of the antigens were confirmed by SDS-PAGE (Figure S2A) and mass spectroscopy (Table S1). Naive Balb/C female mice were immunized with the antigen according to published methods (see details in Methods). After three immunizations, the sera antibody titers bound to wildtype (WT) hIL-1β and E64AcrK reached comparable levels (Figure S2B). The spleens of the immunized mice were isolated for constructing a phage library displaying antibody single-chain variable fragments (scFv).^32^ Next, we designed and performed a “Chemical Crosslinking Panning” (stringent washing steps to remove noncovalent binders, see details in the Methods) against the E64AcrK immunization phage library to identify those phages that can bind to the antigen covalently (Figure 1B). After two rounds of “Chemical Crosslinking Panning,” 28 hits from the output colony forming units (CFUs) were randomly picked and sequenced, resulting in 17 hits with identical scFv amino acid sequences (Figure 1C). This clone was picked to package as a monoclonal phage (designated as CL-E2). When E64AcrK was incubated with the CL-E2 phage in DPBS (pH 8.8) for 48 h, a band corresponding to the molecular weight of E64AcrK+pIII-scFv was detected by Western Blot using antiphage-pIII (Figure 1D,E). To further confirm that the scFv bound to the antigen covalently, we constructed an scFv fusion protein CL-E2-mFc, in which the scFv of CL-E2 was fused with a mouse IgG2a crystallizable fragment (mFc; Figure S2C). When CL-E2-mFc was incubated with E64AcrK under chemical cross-linking conditions, a band corresponding to the covalently cross-linked antigen–antibody complex was also observed (Figure 1F). As a control, CL-E2 or CL-E2-mFc did not cross-link with WT hIL-1β under the same conditions (Figure 1F). In addition, we incorporated AcrK into another site (Q15) of hIL-1β to generate Q15AcrK (Table S1), which did not cross-link with CL-E2-mFc either (Figure 1F). These results demonstrated that this antibody–antigen cross-linking reaction is antibody-epitope orthogonal. It is likely that CL-E2 was clonally selected and evolved in vivo due to the chemical cross-linking activity of AcrK incorporated on the target epitope and was then enriched and identified by our specially designed phage panning method. It is noticeable that the cross-linking reactivity is weak. One possible reason is that our in vitro reaction condition did not exactly mimic the actual condition in the germinal center where B cell selection occurs. Another possible reason is that antigen cross-linking only affords an advantage during clonal selection but not in antibody maturation. Since the antigen covalently binding to BCR already reaches an affinity ceiling, the subsequent antibody maturation will not further improve cross-linking reactivity. Instead, the antibody maturation process may create hits bound to the target epitope independent of cross-linking reactivity. Indeed, both phage CL-E2 and CL-E2-mFc fusion antibodies bound to E64AcrK and WT with similar affinity (Figure S2D,E) under the non-cross-linking conditions. These results suggested that we can utilize a chemical cross-linker to elicit antibody response to a specific epitope and further take advantage of antibody maturation to generate antibodies cross-reactive with the WT antigen in vivo.

### Kcr Incorporated IL1β Induces Epitope-Directed Enrichment of Antibody Responses

**Figure 2:**
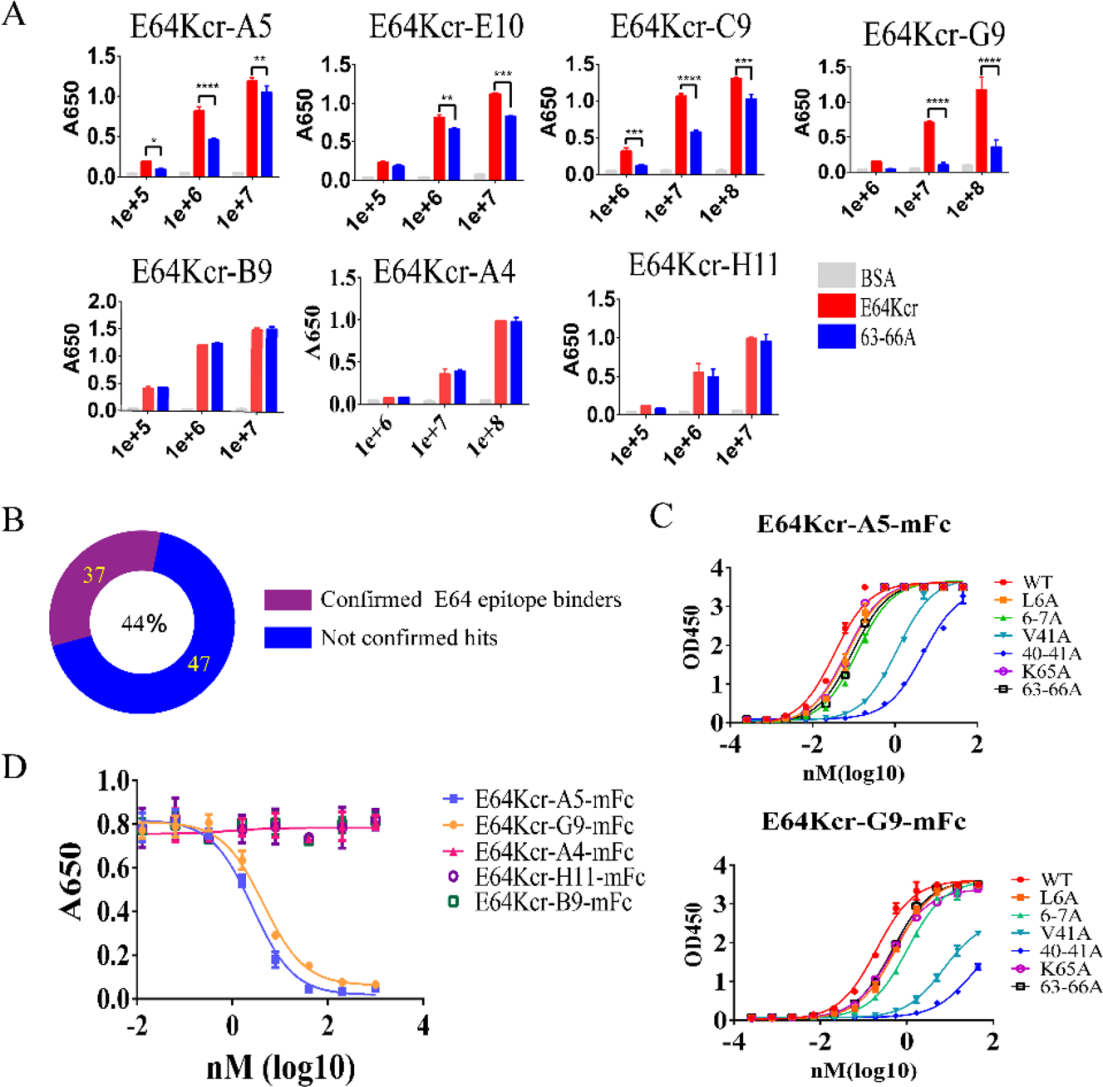
Kcr incorporated IL1β induces epitope-directed antibody responses. (A) Identification of hits bound to hIL-1β E64 epitope based on affinity difference between E64Kcr and hIL-1β63–66A mutant by phage ELISA in pfu titration. The *x* axis is the number of phages (pfu). Each independent ELISA experiment was performed with three technical repeats, the mean value of which is presented as one data column. *p < 0.05; **p < 0.01; ***p < 0.001; ****p < 0.0001. The detected seven hits come from five different clusters, which represent the 84 analyzed hits. (B) The number and percentage of antibodies bound to the E64 epitope based on affinity difference between E64Kcr and the hIL-1β63–66A mutant by phage ELISA. (C) E64Kcr-A5-mFc and E64Kcr-G9-mFc binding affinity with different mutants of hIL-1β. (D) Competition ELISA of five antibodies with canakinumab. The *x* axis is the logarithm of canakinumab concentration. Antimouse IgG antibody was used to detect the amount of E64Kcr-A5-mFc, E64Kcr-G9-mFc, E64Kcr-A4-mFc, E64Kcr-H11-mFc, and E64Kcr-B9-mFc antibodies remaining bound to hIL-1β after canakinumab competition.

To further evaluate the contribution of the chemical cross-linking activity in the epitope directed antibody response, we tested whether Kcr, another lysine analog with weaker chemical cross-linking activity compared with AcrK, can also direct antibody responses to the specific epitope. We constructed, expressed, and purified hIL-1βE64Kcr (Table S1) and performed mouse immunization and phage library construction similarly to those for the AcrK mutant. Next, to evaluate the abundance of antibodies bound to the target epitope in the hIL-1βE64Kcr immunization phage library, we conducted conventional panning (affinity-based selection, normal washing conditions) against hIL-1βE64Kcr. After two rounds of panning, 96 output hits were sequenced and analyzed to yield 84 clones containing full-length mouse scFv which can be grouped into five clusters based on homology (Figure S3). The representative hits from each cluster (cluster I, E64Kcr-A5/E10; cluster II, E64 Kcr-G9/C9; cluster III, E64 Kcr-A4; cluster IV, E64 Kcr-B9; cluster V, E64 Kcr-H11) were picked and packaged as monoclonal phages. To facilitate epitope mapping, we constructed quadruple alanine mutants around residue 64: 63–66 (63–66A).^20^ If the hits bind to the epitope near residue 64, their binding affinities are likely (but not necessarily) distinctive between E64Kcr and the alanine mutants. Gevokizumab, which binds to a distinct epitope^31^ from 63 to 66 with high affinity, showed similar binding affinities to WT hIL-1β and 63–66A (Figure S4), which indicated that 63–66A mutation of hIL-1β had no significant impact on its conformation. We use this ala mutant to quickly profile the selected phage hits. The binding affinities of E64Kcr-A5/E10 (cluster I) and E64Kcr-G9/C9 (cluster II) to hIL-1β63–66A were significantly reduced compared with hIL-1βE64Kcr (Figure 2A), indicating that these two clusters of antibodies likely bind to the E64 epitope of hIL-1β. E64Kcr-A4 (cluster III), E64Kcr-B9 (cluster IV), and E64Kcr-H11 (cluster V) showed no significant affinity difference between hIL-1βE64Kcr and 63–66A, which suggested that the binding of these phages to this epitope may not be through direct interactions with the side chains of residues 63–66 (Figure 2A). However, this does not necessarily rule out the possibility that they could bind to the epitopes nearby. Nevertheless, based on this quick profiling, the monoclonal phages binding to the target epitope accounted for nearly half of the 84 analyzed phage hits (Figure 2B).

Next, we expressed and purified E64Kcr-A5-mFc, E64Kcr-G9-mFc, E64Kcr-A4-mFc, E64Kcr-H11-mFc, and E64Kcr-B9-mFc (Figure S5) to further verify their binding epitopes on the protein level. A more thorough epitope mapping was performed using an alanine-scan method. The binding interface between the antigen and antibody is generally around 900 A^2^;^33,34^ residues in this area may contribute to the binding interactions. We selected the flexible loop region within 10 Å around the E64 site (target epitope) of hIL1b to generate alanine mutants (Figure S6A). Seventeen mutant antigens, including 13 single-alanine mutants and four multialanine mutants, were produced using the same expression and purification method as the WT hIL-1β. Their purities were confirmed by SEC and SDS-PAGE (Figure S6B). Their binding affinities to the aforementioned antibodies were measured and deduced as EC50s by ELISA, respectively (Table S2). The results indicated that residues 6–7, 40–41, and 63–66 of hIL1b are the “hot spots” for the binding of E64Kcr-A5-mFc and E64Kcr-G9-mFc. 40–41A mutations had the most detrimental effects on the binding affinity (Table 1, Table S2, Figure 2C). The key interactions for E64kcr-A4-mFc are similar to those of E64Kcr-A5-mFc and E64Kcr-G9-mFc, except that the 63–66A mutation does not affect the affinity (Table 1, Table S2), which is consistent with the results from the phage binding ELISA. E64Kcr-H11-mFc, a weak binder compared to other hits, showed a different pattern of key interactions, which includes residues 63–66 and 90–91 (Table 1, Table S2). In contrast, none of the selected Ala mutations seemed to affect the binding affinity of E64Kcr-B9-mFc (Table 1, Table S2). In addition, canakinumab was able to compete with E64Kcr-G9-mFc and E64Kcr-A5-mFc to bind hIL-1β (Figure 2D), consistent with the competition phage ELISA results (Figure S7), which can be explained by their overlapped epitope on loop 63–66. Based on these results, E64Kcr-A5-mFc, E64Kcr-G9-mFc, E64Kcr-A4-mFc, and E64Kcr-H11-mFc, which account for 87% of the selected hits, are bound to the regions overlapping or in direct proximity to the target epitope.

**Table 1:** EC50 of Screened Antibodies Binding to Different Antigens, associated with Table S2^a^

	EC50 (nM)				
antigens	E64Kcr-A5-mFc	E64Kcr-G9-mFc	E64Kcr-A4-mFc	E64Kcr-H11-mFc	E64Kcr-B9-mFc
WT	0.034	0.197	0.121	17.41	0.040
L6A	+	+	+	/	/
N7A	/	/	/	/	/
V40A	+	/	/	/	/
V41A	+++	+++	+	/	/
E64A	/	/	/	/	/
K65A	/	+	/	/	/
E64Kcr	/	/	/	/	/
N66A	/	/	/	/	/
V85A	+	/	/	/	/
P87A	/	/	/	/	/
Y90A	/	/	/	/	/
P91A	/	/	/	+	/
V151A	/	/	/	/	/
6–7A	+	++	+	/	/
40–41A	++++	++++	++	/	/
90–91A	+	/	/	+	/
87,90–91A	/	/	/	+	/
63–66A	+	+	/	+	/

a Notes: +, ++, +++, and ++++ mean the EC50 of the corresponding mutant is 2–5-fold, 5–20-fold, 20–50-fold, or in excess of 50-fold higher than WT binding to antibodies, respectively. “/” means the EC50 of the corresponding mutant is less than 2-fold. The actual EC50 values of all mutants are in the Supporting Information (Table S2).

**Figure 3:**
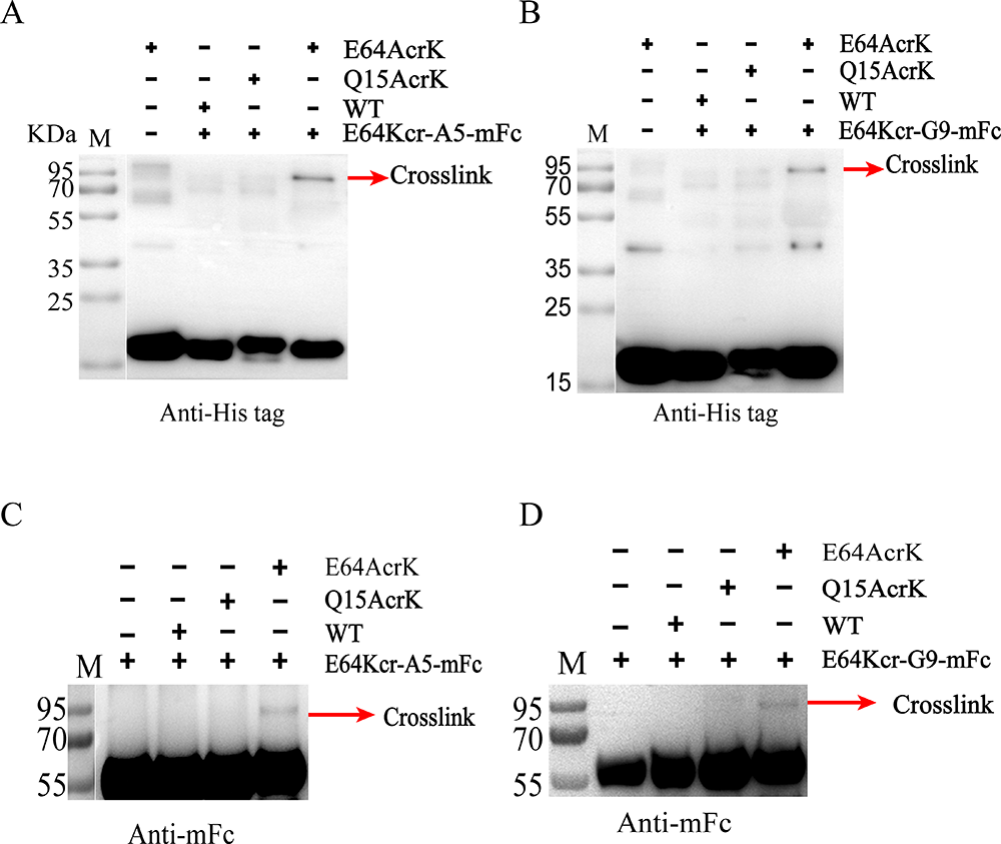
E64Kcr-A5-mFc and E64Kcr-G9-mFc specifically cross-link with E64AcrK. (A and C) Western blot detected E64Kcr-A5-mFc cross-link with E64AcrK by anti-His tag and anti-mFc. (B and D) Western blot detected E64Kcr-G9-mFc cross-link with E64AcrK by anti-His tag and anti-mFc. Antigens were incubated with E64Kcr-A5-mFc or E64Kcr-G9-mFc in a 2:1 molar ratio at pH 8.8 and 37 °C for 2 days (48 h), respectively. The bands corresponding to the cross-linked products are indicated with red arrows.

To find out whether the enrichment of epitope-specific antibodies induced by hIL-1βE64Kcr is due to the chemical reactivity of Kcr, we incubated E64Kcr-G9-mFc or E64Kcr-A5-mFc with hIL-1βE64Kcr under cross-linking conditions for 48 h. No cross-linked product was observed by Western blot analysis (Figure S8). However, incubation of these two antibodies with hIL-1βE64AcrK, which is more reactive than hIL-1βE64Kcr, generated a cross-linked complex (Figure 3). In contrast, these two antibodies did not cross-link with hIL-1βQ15AcrK, an off-site mutant control (Figure 3). The cross-linking reactivity of the hits from hIL-1βE64Kcr is lower than those from hIL-1βE64AcrK, which is corroborated by its lower chemical reactivity. Moreover, all of these hits are cross-reactive with WT hIL-1β (Figure S9), which is consistent with the results of hIL-1βE64AcrK immunization. These results suggested that hIL-1βE64Kcr or hIL-1βE64AcrK can direct the antibody response to the target epitope in vivo by the site-specific cross-linking activity.

### The Epitope-Specific Antibody Response Is Dependent on the Cross-Linking Activity of AcrK and Kcr

In order to exclude the possibility that the elicitation of epitope specific antibody response by hIL-1βE64Kcr and hIL-1βE64AcrK was due to the specific immunogenicity of the E64 epitope, we immunized mice with WT hIL-1β, constructed a phage library, and performed panning using the same method as above. After two rounds of panning, 96 clones were selected randomly from the hit pool for sequence analysis, from which 87 clones containing full-length mouse scFv were identified. Twelve sequence clusters were determined based on homology (Figure S10A). Only one phage clone was identified to bind to the target epitope (Figure S10B). In addition, as a second control, we incorporated *N*ε-Boc-l-lysine (BocK), a ncAA without cross-linking activity, at the same site of the target epitope to generate hIL-1βE64BocK (Table S1) and performed the same immunization and selection experiments. Among the 72 sequences randomly picked from the phage pool after two rounds of panning, only one cluster phage clone seemed to bind to the target epitope (Figure S11A,B). All of the above results demonstrate that the epitope directed antibody response is dependent on the chemical cross-linking activity of AcrK and Kcr incorporated into the target epitope.

### Kcr-Induced Epitope-Specific Antibody Response Is Independent of Epitope Sequence

Our data showed that Kcr or AcrK incorporated antigens induced antibody response to the epitope surrounding E64 efficiently. To investigate whether this mechanism is independent of the epitope sequence, we chose another epitope of hIL-1β for Kcr incorporation. Q15 of hIL-1β is a key site for its interaction with IL1RI.^36^ We constructed, expressed, purified, and characterized hIL-1βQ15Kcr (Table S1) using similar methods as those for hIL-1βE64AcrK. We immunized mice with this mutant, constructed a phage library, and performed two rounds of panning. 96 clones were randomly picked for sequence analysis to yield 89 clones containing full-length mouse scFv. Amino acid sequences of those hits were grouped into 12 clusters (Figure S12). One or two representative sequences from each cluster and three noncluster hits were randomly picked to generate monoclonal phages. To facilitate epitope analysis, the Q15G mutant which can eliminate the binding to IL1RI was generated^35^ (Figure S13A). The affinities of canakinumab and gevokizumab binding to Q15G were nearly the same as their binding affinities with WT hIL-1β, respectively (Figure S13B,C), which indicated that Q15G mutation does not change the overall conformation of hIL-1β. Phage ELISA results showed that all 16 phage clones were cross-reactive with WT hIL-1β (Figure S14A). Eight of them, Q15Kcr-A3, Q15Kcr-B6, Q15Kcr-C1, Q15Kcr-C7, Q15Kcr-D1, Q15Kcr-G8, Q15Kcr-H2, and Q15Kcr-H11, bound to hIL-1βQ15G with significantly reduced affinities compared with hIL-1βQ15Kcr (Figure S14A). These results indicated that the representative hits of eight clusters are bound to the hIL-1βQ15 epitope. It is worth noting that those hits that showed no affinity difference between Q15Kcr and Q15G may still bind to the target epitope as demonstrated in the E64Kcr case previously. Nevertheless, by one simple epitope analysis method, the hits bound to the target epitope already accounted for about 60% of the total number of selected clones if weighting the frequency of each antibody (Figure S14B). We then constructed and purified scFv-Fc fusion protein based on the sequence of Q15Kcr-G8, a representative hit from the cluster with the most abundant sequences (designated as Q15Kcr-G8-mFc; Figure S13D). The binding affinity of this antibody to hIL-1β and hIL-1βQ15Kcr was 3.8 ± 0.9 nM and 2.4 ± 0.6 nM, respectively (Figure S13E). In comparison, its affinity to hIL-1βQ15G was decreased by about 10-fold (Figure S13E), which is consistent with the phage ELISA results (Figure S14A). Together, these results suggested that a Kcr induced epitope-directed antibody response is independent of the epitope sequence context.

### Kcr Incorporated IL-1β Elicits Neutralization Antibody Titer in Vivo

Several IL-1β based constructs have been evaluated for their potential as vaccines; however, the efficacy has yet to be demonstrated in clinical trials.^36−38^

**Figure 4:**
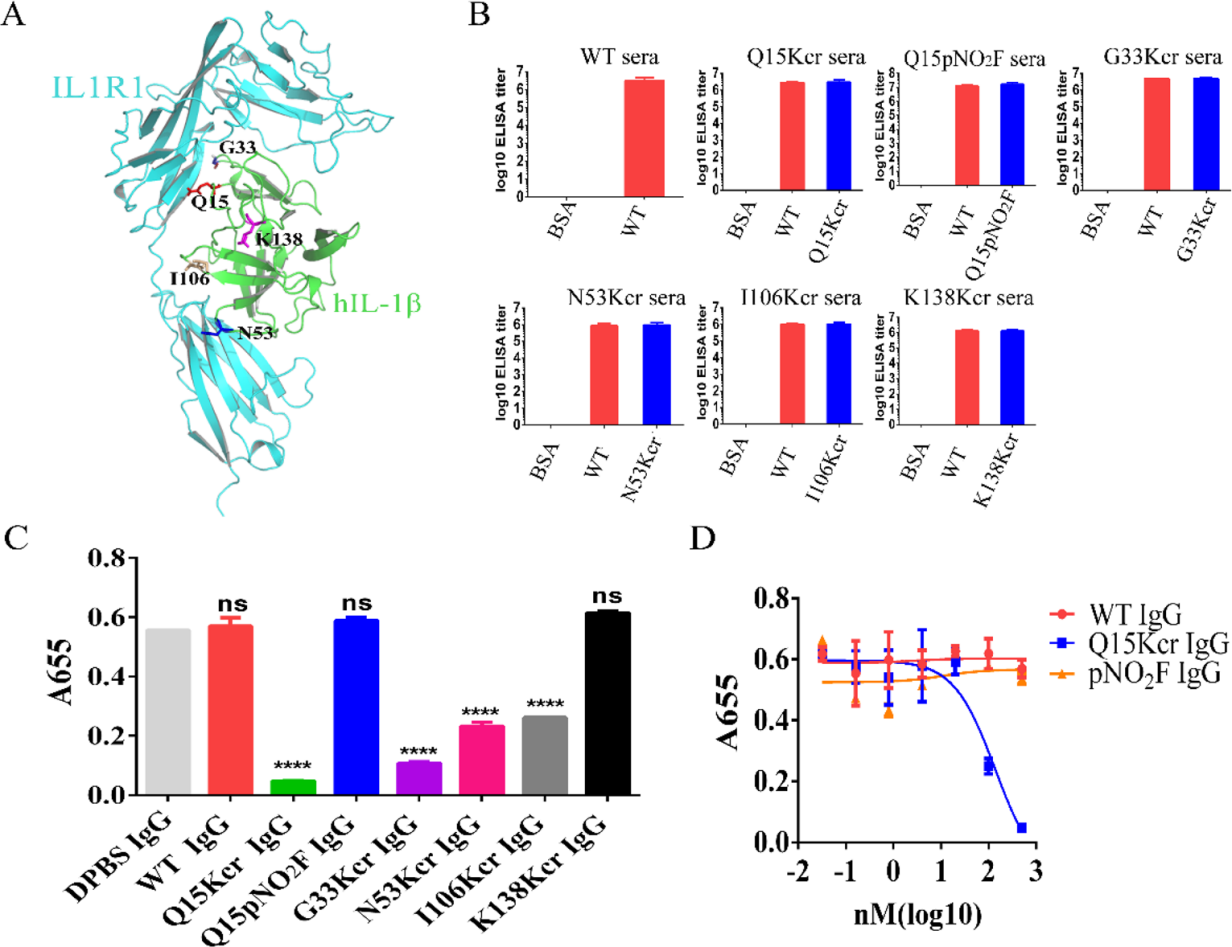
Antibodies elicited by Kcr incorporated epitope exerted hIL-1β neutralization effect. (A) Structure of the hIL-1β complex with IL1RI. G33, Q15, I106, and N53 of hIL-1β are in the interface of the complex, while K138 is far from that interface. (B) Serum titers of mice (*n* = 3) immunized with WT hIL-1β and ncAA-incorporated mutants. Antibody titers are expressed as the reciprocal of the serum dilutions needed to achieve half-maximal absorbance in ELISA. (C) Neutralization assay of total IgG from WT hIL-1β, Q15Kcr, G33Kcr, N53Kcr, I106 Kcr, K138Kcr, and Q15pNO_2_F immunized mice. HEK-Blue IL-1R was used as the reporter cell line. The secreted SEAP in the supernatant was detected by QUANTI-Blue. Each independent hIL-1β inhibition experiment was performed with three technical repeats and three biological repeats, which had similar results, and one repeat result is shown in the figure, the mean value of which is presented as one data column. ****p < 0.0001; ns, not significant. Each column was compared with the DPBS group. (D) Dose-dependent inhibitory activities of total IgG from Q15Kcr, WT, and Q15pNO_2_F immunized mice.

Although the antibody response of conventional subunit vaccines can be enhanced by engineering the constructs and/or combination with adjuvants, the ratio of the neutralization titer to the total antibody titer is generally low and difficult to increase by current methods. We reason that the enrichment of epitope-directed antibodies elicited by the Kcr incorporated antigen would neutralize the function of IL-1β efficiently in vivo if the target epitope is located at the key binding interface with IL1RI. According to the structure of the hIL-1β-IL1RI(ECD) complex (Figure 4A),^39^ Q15, G33, N53, and I106 are located on the interface, and therefore these positions were selected to incorporate Kcr, respectively, to afford the corresponding mutants (designated as G33Kcr, N53Kcr, and I106 Kcr based on the site of amino acid substitution; Table S1). Mice were immunized with these mutants according to published methods.^40^ The serum IgG titers were examined 10 days after the third immunization. The mice immunized with the WT hIL-1β and the mutants showed high and comparable titers (∼1:10^6^, Figure 4B). Next, we collected the mouse sera, purified the total IgG by protein A resin (Figure S15), and evaluated the neutralization effect of the total IgG from each group. HEK-Blue IL-1R is an engineered cell line that stably expresses IL1RI on the cell membrane and can be activated by IL-1β to secrete embryonic alkaline phosphatase (SEAP). The IL-1β level can be evaluated by measuring the amount of SEAP in cell culture.^41^ The total IgG from Q15Kcr, G33Kcr, N53Kcr, and I106 Kcr immunized mice significantly inhibited the activation of HEK-Blue IL-1R cells by hIL-1β, while the IgG from WT and DPBS immunized mice had no inhibitory effect (Figure 4C, Figure S16). Among those, the IgG from hIL-1βQ15Kcr immunized mice had the strongest inhibition effect (Figure 4D). As a control, a K138 Kcr mutant was generated (Table S1), in which Kcr was incorporated at the non-IL1RI binding site residue 138 (Figure 4A). As expected, the total serum IgG from K138Kcr immunized mice did not inhibit the activation of HEK-Blue IL-1R cells by hIL-1β under the same immunization and assay conditions (Figure 4C, Figure S16). Furthermore, as a second control, pNO_2_F was incorporated to replace Q15 to yield Q15pNO_2_F (Table S1). IgG produced from mice immunized with this mutant did not show neutralization activity either (Figure 4C,D), even though its total titer was similar to that of the Kcr mutant (Figure 4B). These results corroborated the previous observations that pNO_2_F incorporated TNFα showed enhanced total antibody titer but not specifically to the incorporated epitope.^21,24^

### Kcr Modified Peptides Also Elicit Epitope-Directed Antibody Responses

**Figure 5:**
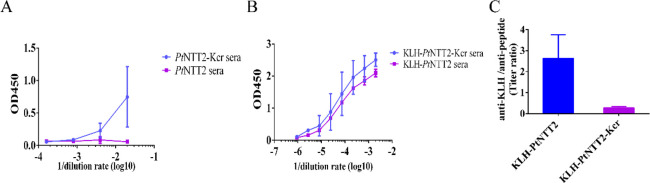
Kcr modified peptides elicit antibody enrichment. (A) The sera titer of *Pt*NTT2-Kcr and *Pt*NTT2 peptides immunized mice against corresponding peptides. (B) The sera titer of KLH-*Pt*NTT2-Kcr and KLH-*Pt*NTT2 immunized mice against corresponding peptide-KLH conjugates. (C)The ratio of sera titer binding to KLH versus peptide from KLH-*Pt*NTT2-Kcr and KLH-*Pt*NTT2 immunized mice.

Conjugation of peptide or protein antigens to Keyhole Limpet Hemocyanin (KLH) is a common approach utilized to enhance the immunogenicity of the antigens, which usually increases antibody titers during immunization. However, the elicited antibodies are more likely to bind to the KLH than the target antigens with much smaller sizes.^42−45^ Based on the proposed mechanism described above, we hypothesize that the epitope-directed antibody enrichment caused by Kcr may also help to enhance antibody responses to the target peptide in the context of the peptide-KLH conjugate. *Pt*NTT2 exerts a crucial role in the counter exchange of nucleoside triphosphates through the outer membrane from the cytoplasm tostromata.^46^ The extracellular domain of *Pt*NTT2 selectively recognizes different nucleoside triphosphates and their analogs. Antibodies bound to the selective epitope of this protein may help to study its structure and function. We designed and synthesized the wildtype peptide (AKPAADNEQSIKPKKKKPKM) derived from the extracellular domain of *Pt*NTT2 and its K12Kcr mutant (AKPAADNEQSIKcrPKKKKPKM). Mice were immunized with these two peptides. After three rounds of immunization, serum titer analysis showed that *Pt*NTT2-Kcr provoked weak antibody responses, while WT *Pt*NTT2 did not elicit any antibody titer (Figure 5A). To increase the immunogenicity and the titer of antibody responses, KLH-*Pt*NTT2 and KLH-*Pt*NTT2-Kcr were generated by conjugation of peptide and KLH and used to immunize mice. Both antigens induced comparable antibody titers against the conjugated immunogen (Figure 5B). Interestingly, KLH-*Pt*NTT2-Kcr induced 6-fold higher antibody titer binding to the *Pt*NTT2-Kcr peptide than that to KLH (Figure 5C). In contrast, the majority of the antibody titer induced by KLH-*Pt*NTT2 bound to KLH (Figure 5C). The *Pt*NTT2 peptide only contains 20 amino acids, less than 1% of the length of KLH (3400 aa), and is a much weaker immunogen compared to KLH. By incorporation of a single residue, Kcr, with chemical cross-linking activity, a significant portion of the antibody responses were enriched to the target peptide. These results indicated that Kcr incorporation created an immunodominant epitope in the peptide and elicited epitope-directed antibody responses in vivo, which is consistent with the results from the study of Kcr incorporated hIL-1β. These results not only further demonstrated the mechanism of chemical cross-linking activity in the elicitation of epitope-directed antibody responses but also enabled its utility in enhancing the antibody titer to the target peptide in conjugation with other strong immunogens.

## Discussion

A few ncAAs with chemical reactive side chains such as AcrK, Kcr, FpheK, and fluorosulfate-l-tyrosine (FSY) have been incorporated into proteins and enabled their cross-linking with binding partners in proximity.^25,26,47,48^ The process requires screening for or engineering of a suitable site in vitro to allow the reaction to occur. Here, we incorporated AcrK and Kcr into the target site to create the chemically active antigen and immunized mice to generate antibodies that could cross-link with the antigen. By taking advantage of antibody selection and maturation processes in vivo, coupled with a customized phage panning method, antibodies that bind covalently with the target antigen can be identified. A hapten carrying a reactive small molecule has been used to generate cross-linking antibodies, where the reaction site is within the antibody binding pocket.^49,50^ In contrast, we generate orthogonal antibody–antigen cross-linking by in vivo antibody evolution and create specific reaction sites on the protein–protein binding interface.

Based on these results, we developed a new approach for facile elicitation and identification of antibody binding to a specific epitope of an antigen. Antibodies produced from mouse immunization of a whole antigen are usually enriched to immune-dominant B-cell epitopes, often diminishing the probability of identifying antibodies binding to the desired epitopes. By incorporating AcrK or Kcr into the target site on the IL-1β, “super” immune-dominant B cell epitopes can be created, and antibody responses were directed and enriched to the target epitopes after mouse immunization. This effect is so prominent that that the majority of the randomly picked hits from IL-1βE64AcrK and IL-1βE64Kcr immunization bind to the target epitope. In contrast, very few hits were identified to bind to the target epitope by immunization with WT IL-1β and mutants incorporated with non-cross-linking ncAAs.

More importantly, the epitope-directed antibody response to Kcr is not restricted by amino acid sequence. On multiple sites of IL-1β, Kcr could effectively induce high titers of epitope specific antibodies. When the target epitope is located on the interface between IL-1β and IL1RI, the epitope specific antibodies elicited by Kcr effectively block IL-1β to activate its receptor. Therefore, this epitope-directed antibody response can be applied to vaccine development to enhance effective antibody responses to the functional epitopes. Moreover, this mechanism and method also apply to peptide-KLH conjugates, which enriched more than half of the total sera titer to the Kcr incorporated small peptide with low immunogenicity.

Previously, another ncAA, pNO_2_F, without chemical cross-linking activity was explored for a similar purpose. pNO_2_F incorporated antigens were shown to enhance total titer but not epitope-directed titer. It is believed that its nitroaryl group with an electron-deficient π system can interact with the Tyr and Trp side chains common to germline antibodies. And it was suggested that breaking both T and B cell tolerance increased the total antibody titer.^21,24^ Therefore, pNO_2_F incorporated in the antigen functions more similarly to an adjuvant to boost the whole antibody response but is not epitope-directed.

The functional epitope typically only accounts for a very small surface area of the whole antigen and is often masked by nonfunctional but immune-dominant B cell epitopes. Current methods can only increase the total titer of antibody responses to the antigen by adding adjuvants or optimizing the antigen particle size^51−53^ but cannot selectively enhance the effective titer needed to exert functionality. Our method represents a breakthrough of this key bottleneck, which has great potential in the selection of therapeutic antibodies for the “difficult” targets and the development of protein subunit vaccines.

## Methods

### Mice

All procedures with mice were reviewed and approved by the Academy of Sciences. Six to eight week old female Balb/C mice were used in the studies. Mice were housed in the animal facility under SPF conditions with a 12 h light/dark cycle at room temperature in accordance with the institutional guidelines and ethical regulations and fed with regular chow and water. Mice were randomly assigned to experimental groups.

### Expression and Purification of hIL-1β and Its Mutant

To overexpress WT hIL-1β and alanine/glycine mutants, *E. coli* BL21 (DE3) competent cells were transformed with the corresponding plasmids in the pET28a vector. After the Sanger sequence verified the expression vectors, a correct clone was inoculated into 2 X YT medium supplemented with kanamycin (50 μg/mL) at 37 °C. When the growth of bacteria’s OD600 reached 0.6, 0.5 mM isopropyl-β-d-thiogalactopyranoside (IPTG) was added to induce expression at 18 °C overnight. To overexpress AcrK or Kcr incorporated mutants of hIL-1β, *E. coli* BL21 (DE3) competent cells were cotransformed with pEVOL-MmPrKRS or pEVOL-MmBukRS and the corresponding hIL-1β expression plasmid containing the amber codon (TAG). Expression strains were cultured in 2 X YT medium supplemented with kanamycin (50 μg/mL) and chloromycetin (25 μg/mL). The cells were allowed to grow for 3 to 5 h; when the OD600 reached 0.8, 1 mM IPTG, 5 mM Kcr or 10 mM AcrK, and 0.2% l-arabinose (m/v) in final concentrations were added to induce ncAA incorporated protein expression. As for the expression of BocK or pNO_2_F incorporated mutants, the respective orthogonal plasmid is pUltra-pNO_2_RS^54^ or pDule-pylRS.^55^ The rest of the procedures are the same as those of the Kcr incorporated mutant. The induced expression strains were grown for an additional 15 h at 30 °C before harvesting by centrifugation at 6000*g* for 10 min. The cell pellets were lysed by sonication, and the cell lysate was clarified by centrifugation at 13 000*g* for 30 min at 4 °C. WT hIL-1β and mutants were purified on Ni-NTA resin (GE Healthcare, 17–0575–01) following the manufacturer’s instructions. The proteins were further purified through a Superdex 75 increase 10/300 GL column (GE Healthcare, 10263259) in DPBS buffer. Purified protein aliquots were flash frozen with liquid nitrogen and stored at −80 °C until use.

### Mouse Immunization

Female Balb/C mice (*n* = 3 per group), 6–8 weeks old, were randomly administrated subcutaneously with WT hIL-1β or UAA incorporated mutants. For the first immunization, 50 μg of antigen was mixed with Freund’s complete adjuvant (Sigma, F5881). The mice were then immunized two more times to boost, 2 weeks apart, with 30 μg of antigens mixed with Freund’s incomplete adjuvant (Sigma, F5506).

### Expression and Purification of scFv-mFc Fusion Proteins

The DNA fragment of scFv fused with mouse IgG2a Fc by a glycine-serine linker^46^ was ligated into the linearized pFuse expression vector. HEK 293F cells (Thermo Scientific, R79007) were cultured in shaker flasks containing FreeStyle medium (Thermo Scientific, 12338026) and shaken at 125 rpm at 37 °C, with 5% CO_2_. Then, 2.5 × 10^6^ cells/mL were transfected with the scFv-mFc expression plasmid mixed with PEI at a ratio of 1:2.5 (W/W). The expression media were harvested when the cell viability decreased to below 75%. The collected supernatant was loaded onto a column with Protein A resin (GenScript, L00210) twice, which was pre-equilibrated in DPBS. After washing with 10 column volumes of DPBS, the protein sample bound to the resin was eluted with elution buffer (0.2 M glycine, 0.1 M NaCl, pH 2.5). Immediately after elution, Tris-HCl (100 mM final concentration) was added to adjust the pH to 7.5. The eluted protein was then concentrated using Amicon Ultra centrifugal filters (Merck Millipore, UFC903096), and the buffer was exchanged with DPBS (pH 7.5) and further purified by size-exclusion chromatography using a Superdex 200 increase 10/300 GL column (GE Healthcare, 10263259). Purified protein aliquots were flash frozen by liquid nitrogen and stored at −80 °C until use.

### Mouse Total IgG Purification

After three immunizations, mouse sera were collected and diluted with an equal volume of DPBS (pH 8.0). Samples were incubated with protein A resin (GenScript, L00210) and then washed with 10 column volumes of DPBS. The protein bound to the protein A resin was eluted with elution buffer (0.2 M glycine, 0.1 M NaCl, pH 2.5). Immediately after elution, Tris-HCl (100 mM final concentration) was added to adjust the pH to 7.5. The eluted IgG was then concentrated using Amicon Ultra centrifugal filters (Merck Millipore, UFC903096) and exchanged buffer with DPBS (pH 7.5). Purified protein aliquots were flash frozen by liquid nitrogen and stored at −80 °C until use.

### Enzyme-Linked Immunosorbent Assay (ELISA)

The antigen (100 ng) was coated on a 96-well ELISA plate (Corning Costar, 2592) at 4 °C overnight, after which the plate was blocked with 200 μL of 3% skim milk solution in DPBS for 2 h at 37 °C. Antibodies or phages were added with 3% skim milk in DPBST (0.05% Tween20) for 2 h at 37 °C. Wells were washed four times with 200 μL of DPBST. Subsequently, horseradish peroxidase (HRP) conjugated detection antibody was added to the blocking solution and incubated for 1 h at RT. Wells were then washed five times with 200 μL of DPBST. A 100 μL working solution of trimethylboron (TMB; Biolegend, 002023) was added to each well and incubated for 10 to 30 min at RT before A650 was read using a plate reader (BMG LABTECH, CLARIOstar Plus). If the OD450 value was adopted, the final step of the chromogenic reaction was quenched by 50 μL of 1 M sulfuric acid for 10 min.

Competition ELISA: 100 ng WT hIL-1β was coated on the ELISA plate at 4 °C overnight. Wells were blocked with 3% BSA in DPBS for 2 h at 37 °C, and a series of concentrations (start at 1 μM, 5-fold dilution) of canakinumab were added (in 3% BSA, 0.05% Tween 20) for 1 h at RT. After 1 h of incubation, 0.2 nM E64Kcr-A5-mFc, 10 nM E64Kcr-G9-mFc, 10 nM E64Kcr-A4-mFc, 10 nM E64Kcr-B9-mFc, or 10 nM E64Kcr-H11-mFc were added to incubate for another 1 h. After washing, HRP-conjugated goat antimouse IgG Fc antibody was added at a dilution of 1:5000 in blocking solution and incubated for 1 h at room temperature. A 100 μL working solution of trimethylboron (TMB; Biolegend, 002023) was added to each well and incubated for 10 to 30 min at room temperature before plates were read using a plate reader (BMG LABTECH, CLARIOstar Plus).

### Surface Plasmon Resonance (SPR)

Gevokizumab (20 μg/mL) was immobilized onto the four individual flow cells in the CM5 sensor chip by a standard amino coupling protocol using Biacore T100. The antigens were 2-fold series diluted from 50 nM to 3.125 nM, which were injected into the T100 system from low to high concentration. A blank buffer for baseline subtraction was sequentially injected, with a regeneration step inserted between each cycle. The antibody surface was regenerated with two 15-s pulses of glycine (pH 2.0). The binding interactions were monitored over a 60 s association period and a 600 s dissociation period (running buffer only). The binding kinetics curves were processed by Biacore software.

### Western Blot

Samples were mixed with loading buffer which contained 20 mM DTT and 2% SDS. After heating at 95 °C for 10 min, samples were subjected to SDS-PAGE on a polyacrylamide gel. The gels were subsequently transferred onto a polyvinylidene difluoride filter (PVDF) film (Bio-Rad, 1620177) using biorad trans-blot at a 300 mA constant current. After completing the film transfer, it was blocked with 5% skim milk in DPBS for 2 h and then incubated at room temperature for 2 h with the first antibody in the blocking solution. Membranes were washed four times in DPBST, then incubated at room temperature for 1 h with 1:5000 diluted secondary antibody (HRP conjugated) in blocking solution and finally washed with DPBST four times. Signals were generated by using an enhanced chemiluminescence (ECL) reagent (Thermo Fisher Scientific, 35055) and detected with a Tanon 5200 system.

### Total RNA Extraction and Reverse Transcription from Mouse Spleen

Two weeks after the completion of the third immunization, the spleens of mice were isolated, and a portion of spleen tissue (about 100 mg) was quickly frozen with liquid nitrogen and ground until it became a powder in a low-temperature environment. Then, 1 mL of Tirzol (Invivogen, NO.15596018) was added to fully lyse the tissue cells, followed by the following steps: (1) Add 200 μL of chloroform to the lysed solution. Mix completely at room temperature, and keep still for 3–5 min. (2) Centrifuge at 12 000 rpm for 10 min at 4 °C, and transfer the supernatant to a 1.5 mL EP tube containing 0.5 mL of precooled isopropanol (no RNA Enzyme), mixed completely, and placed at room temperature for 10–30 min. (3) Centrifuge at 12 000 rpm for 10 min at 4 °C, Decant the supernatant. Wash twice with 75% ethanol, 1 mL each time, and dry in the biosafety cabinet. (4) Add 50 μL of sterile DEPC-treated ddH_2_O to dissolve total RNA. (5) Agarose gel electrophoresis was used to detect RNA purity, and NanoDrop was used at the same time to quantify the concentration of the total RNA.

Reverse transcription: The cDNA was generated by a reverse transcription kit (Thermo Scientific, No. 00644497) according to its provided standard protocol.

### Construction of Phage Display Library

Phage display libraries were constructed using the published methods.^32^ The cDNA obtained by reverse transcription was used as a template for the amplification of VH and VL of mouse antibodies with heavy and light chain primers reported in the literature.^56^ The VH and VL were amplified by overlap PCR to obtain antibody scFv fragments. The amplified scFv product and phage display plasmid pSEXRTL2 were digested with a special restriction enzyme Sfi I, and the digested scFv fragment and linear vector were ligated with T4 ligase (NEB, No.10083330). Subsequently, the ligation production was purified and dissolved in ddH2O using a DNA Extraction Kit (Qiagen, No. 28706 × 4). The purified ligation product was transformed into a freshly prepared XL1-Blue electroporation-competent cell. After electroporation, the cells were incubated at 220 rpm for 1 h at 37 °C and spread on 2 X YT plates (Amp and Tet dual antibiotics), growing overnight. At the same time, part of the bacterial solution from electroporation was taken for colony counting by the gradient dilution method, according to the Formation of Colonies Unit (CFU) computer library capacity. The next day, 96 colonies were randomly selected, and scFv fragments were amplified by colony PCR to verify the clone containing an scFv fragment. The positive rate was counted, and the positive clones were selected for Sanger sequencing to further detect the quality of the library. After that, all of the bacterial colonies on the plates were collected with a cell scraper, and 20% sterile glycerol was added and mixed and then divided into 1 mL aliquots stored at −80 °C.

### Phage Production

*E. coli* XL1-Blue cells carrying the phagemids (displaying scFv-pIII) were inoculated in 20 mL of 2 X YT medium with ampicillin (100 μg/mL) and tetracycline (15 μg/mL) and cultured at 37 °C and 220 rpm. When OD600 reached 0.5, 20 multiplicities of infection (MOI) of M13KO7 (ΔpIII) hyperphage (Progen, No. PRHYPE) was added to infect cells at 37 °C and 120 rpm for 1 h. The infected cells were spun down and resuspended in 40 mL of 2 X YT medium with ampicillin (100 μg/mL), tetracycline (15 μg/mL), and kanamycin (50 μg/mL) at 30 °C and 250 rpm for another 13 h. The overnight-growth culture was centrifuged at 4000*g* for 10 min. The supernatant was transferred to a new tube and centrifuged at 10 000*g* for 20 min. The 5 X phage precipitating buffer [polyethylene glycol 8000 (PEG 8000)/NaCl: 100 g of PEG 8000, 73.3 g of NaCl dissolved in 500 mL ddH_2_O] was added and placed on ice for 4 h. Phages were harvested by centrifugation at 10 000*g* at 4 °C for 20 min and solubilized using 1 mL of DPBS. Another centrifuge was conducted to remove the remaining bacteria. The purified phage was kept at 4 °C for a week and showed no titer decrease. For long-term storage, 10% sterile glycerol was added to the phage solution, flash frozen using liquid nitrogen, and stored at −80 °C for up to 6 months with no obvious titer decrease.

### Phage Panning

Conventional phage panning: The antigen (1 μg) was coated on plate wells in DPBS overnight at 4 °C, which were then blocked with 200 μL of 3% skim milk at room temperature for 2 h. After washing wells twice with DPBST, approximately 10^10^ pfu phages from the immune libraries of WT hIL-1β, E64Kcr, E64BocK, or Q15Kcr were added and incubated at RT for 2 h. The wells were then washed with DPBST 10 times, 3 min each time. After washing, the bound phages were eluted by incubating with 1 mg/mL trypsin (Gibco) for 20 min at RT. The collected phages were used for the next round of panning.

Chemical cross-linking phage panning: E64AcrK (1 μg) was coated on plate wells in DPBS overnight at 4 °C, which were then blocked with 200 μL of 3% BSA (dissolved in DPBS) at RT for 2 h. Approximately 10^10^ pfu phages (dissolved in DPBS, 1 mM EDTA, pH 8.8) were added and incubated at 37 °C for 48 h. The wells were washed under stringent conditions, including two times by DPBS containing 10 mM DTT (total of 5 min), 10 times by DPBST (total of 20 min), twice by 0.15% SDS ddH_2_O solution (total 3 min), 10 times by DPBS (total 20 min), once by an acidic buffer (0.2 M Glycine, pH 2.2; total 3 min), 10 times by DPBST (total 20 min), and twice by DPBS (total 5 min). After washing, the bound phages were collected by incubating with 1 mg/mL trypsin (Gibco) for 20 min. Collected phages were used for infecting *E. coli* XL1-Blue to produce phages, then the next round of chemical cross-linking panning proceeded.

### Chemical Cross-Linking Reaction

Eight micromolar E64AcrK, Q15AcrK, E64Kcr, and Q15Kcr were incubated with 4 μM corresponding antibodies (CL-E2-mFc, E64Kcr-A5-mFc, and E64Kcr-G9-mFc) under DPBS conditions (containing 1 mM EDTA, adjust pH to 8.8) and 37 °C for 2 days. For phage chemical cross-linking, 10^8^ pfu phages (CL-E2) were incubated with 8 μM E64AcrK under the same alkaline conditions for 2 days. All reactions were maintained under sterile conditions.

### hIL-1β Neutralization Assays

HEK-BlueTM IL-1R cells (Invivogen, hkb-il1r) at 70% confluence were washed twice with prewarmed PBS, then the cells were detached in the presence of PBS by tapping the flask. Cells were resuspended in fresh, prewarmed DMEM (contain 10% heat-inactivated FBS) at ∼330 000 cells/mL. In 96 separate wells, 25 μL of a recombinant human IL-1β (0.8 ng/mL) was incubated with 25 μL of purified sera IgG in a 1:5 dilution series starting at a concentration of 4 μM in DPBS at room temperature for 30 min. A total of 150 mL of HEK-Blue IL-1R cell suspension (∼50 000 cells) was added per well. The 96-well plate was incubated overnight at 37 °C in 5% CO_2_. Twenty microliters of cell culture supernatant was transferred and incubated with 180 μL of QUANTI-Blue (Invivogen) per well in the flat-bottom 96-well plate at 37 °C for 30 min to 3 h. The secreted embryonic alkaline (SEAP) was then detected using a plate reader (CLARIOstar Plus) at 655 nm.

### ScFv Sequence Analysis

All scFv amino acid sequences were aligned using ClustalW (MEGA-X; DNA weight matrix, IUB; gap opening penalty, 15.00; gap extension penalty, 6.66), and the maximum-likelihood phylogenetic tree was calculated using MEGA-X with 1000 bootstrap replicates. The scFv amino acids of hits used for phage ELISA can be found in the Excel file of scFv sequences.

### Peptides Synthesis and Conjugation with KLH

*Pt*NTT2, AKPAADNEQSIKPKKKKPKM, and *Pt*NTT2-Kcr which replaced the K12 to Kcr, AKPAADNEQSIKcrPKKKKPKM, were synthesized by Hubei Qiangyao Biotechnology Co. Ltd, China. The synthesized peptides were conjugated with KLH by the same company.

### Quantification and Statistical Analysis

ELISA data were compared by two-way ANOVA, followed by multiple comparisons using Prism 6.0 (GraphPad software). All of the *P* values were calculated using GraphPad Prism 6.0 with the following significance: n.s., *p* > 0.05; *p < 0.05; **p < 0.01; ***p < 0.001; and ****p < 0.0001. Statistical details for each experiment can be found in the figures and the legends.

## Supporting Information Available

Supplemental Figures S1–S16 and Tables S1 and S2l Table S1 shows the ESI-MS data of different ncAA incorporated protein; Table S2 shows the EC50 value of selected antibodies binding to hIL-1β and its mutants (PDF)
scFv sequences (XLSX)
