# Supplementary material for: Epitope-Directed Antibody Elicitation by Genetically Encoded Chemical Cross-Linking Reactivity in the Antigen
Source: ACS Cent Sci. 2023 Jun 6;9(6):1229–40. doi: 10.1021/acscentsci.3c00265 (PMC10311653; doi:10.1021/acscentsci.3c00265)
Supplement: Supplementary file 1 — oc3c00265_si_001.pdf [file oc3c00265_si_001.pdf]

Supplementary Materials for

Epitope-directed antibody elicitation by genetically encoded crosslinking reactivity in the antigen

Chaoyang Zhu,[1,2,3 #] Liang Xu,[1,2 #] Longxin Chen,[1,4] Zihan Zhang,[1,5] Yuhan Zhang,[1] Weiping Wu,[6] Chengxiang Li,[1,2] Shuang Liu,[1,2] Shuqin Xiang,[1,2]Shengwang Dai,[1,2] Jay Zhang,[1,6] Hui Guo,[1,6,7] Yinjian Zhou,[1,7] and Feng Wang[1,6,7*]

[1]Key Laboratory of Protein and Peptide Pharmaceutical, Institute of Biophysics, Chinese Academy of Sciences, Beijing 100101, China.

[2]College of Life Sciences, University of Chinese Academy of Sciences, Beijing 100101, China.

[3]Current address: The Texas A&M Drug Discovery Laboratory, Department of Chemistry, Texas A&M University, College Station, TX, 77843, USA.

[4]Molecular Biology Laboratory, Zhengzhou Normal University, Zhengzhou 450044, China.

[5]Current address: Institute of Biosciences and Technology, Texas A&M Health Science Center, Houston, TX, 77030, USA.

[6]Suzhou Institute for Biomedical Research, Suzhou, Jiangsu 215028, China.

[7]Beijing Translational Center for Biopharmaceuticals, Beijing 100101, China.

[#] These authors contributed equally.

[*]Corresponding author. Email: wangfeng@ibp.ac.cn

This PDF file includes:

Figs. S1 to S16
Supplemental Table S1, S2

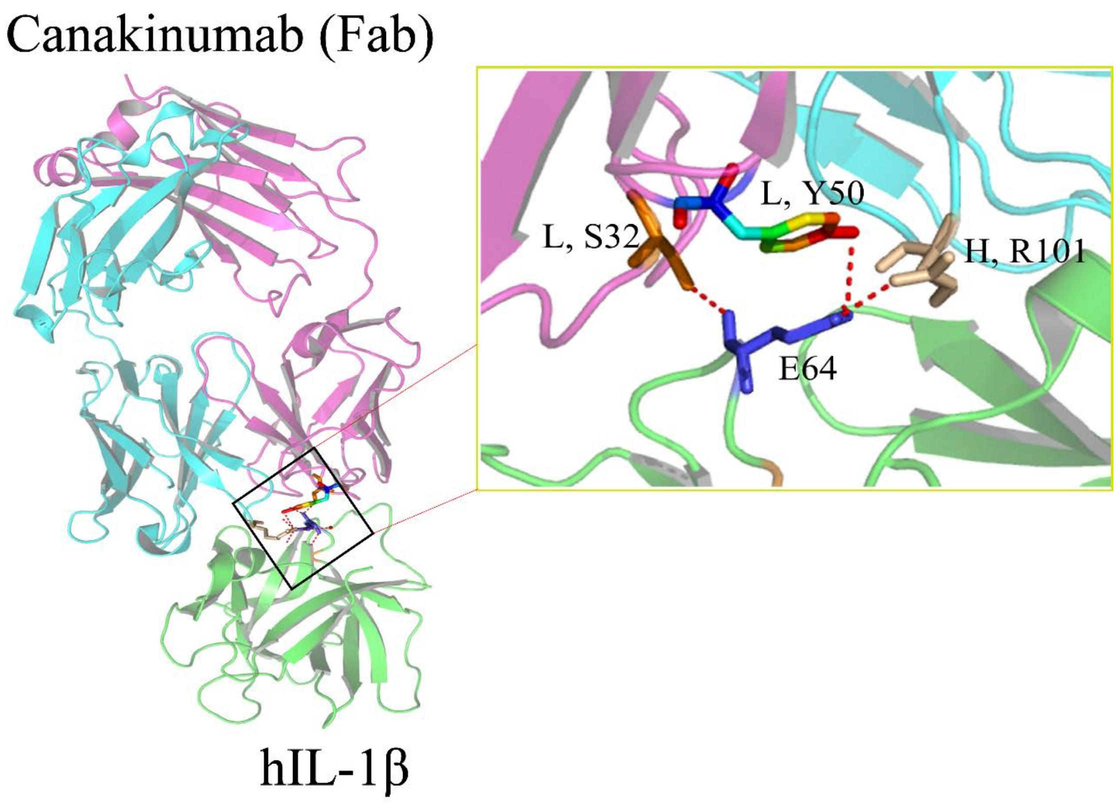

Figure S1. Based on the hIL-1β-Canakinumab Fab complex (PDB: 4G6J), residue E64 of hIL-1β interacts with the residue S32 (L, S32) and Y50 (L, Y50) in the light chain, and R101(H, R101) in the heavy chain of canakinumab (Fab).

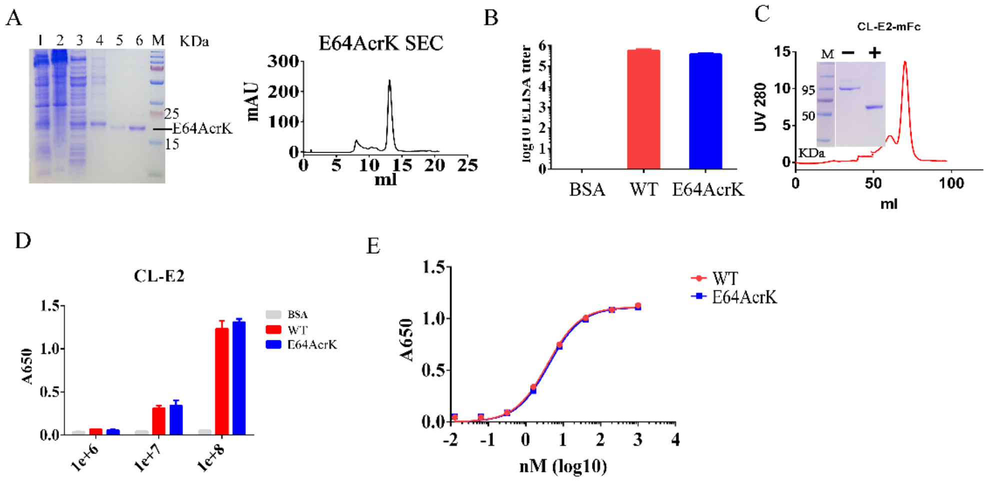

Figure S2. (A) Expression and purification of E64AcrK. **(B)** Serum titers of the mice immunized with E64AcrK. Titers are expressed as the reciprocal of the serum dilutions needed to achieve half-maximal absorbance in ELISA. **(C)** The purified CL-E2-mFc fusion antibody was analyzed by SEC and SDS-PAGE. -, without DTT; +, with 10 mM DTT. **(D)** Phage CL-E2 bound to WT hIL-1β and E64AcrK with similar affinity**. (E)** Fc fusion antibody CL-E2-mFc bound to WT hIL-1β and E64AcrK with similar affinity.

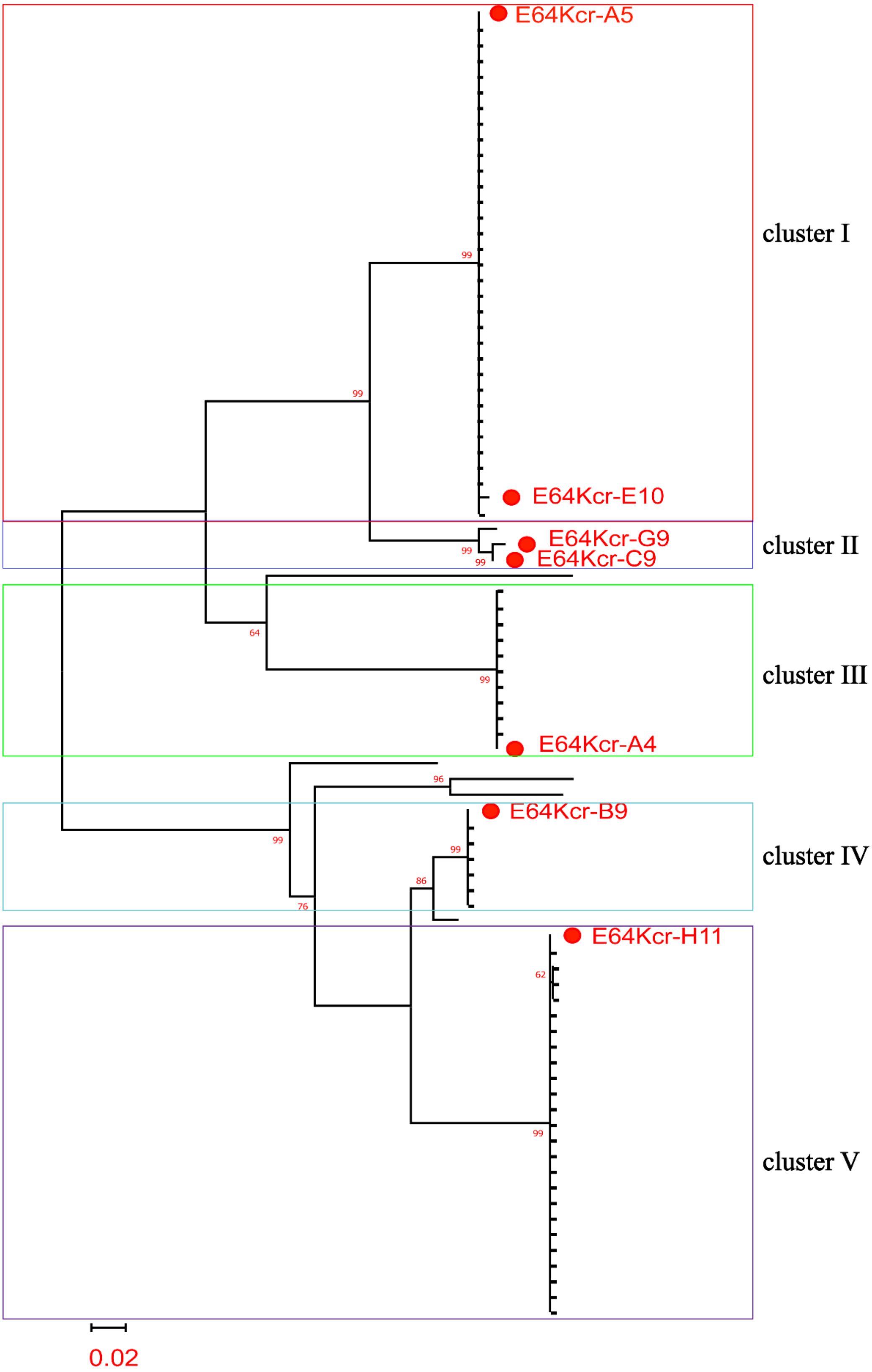

Figure S3. Sequence analysis of the output clones from panning E64Kcr immunization phage library. 84 clones containing correct mouse scFv sequences were grouped into five clusters based on their amino acid sequence homology. The solid red circle represents clones selected for antigen binding epitope verification.

Model: 1:1 Binding **Temperature** : 25℃

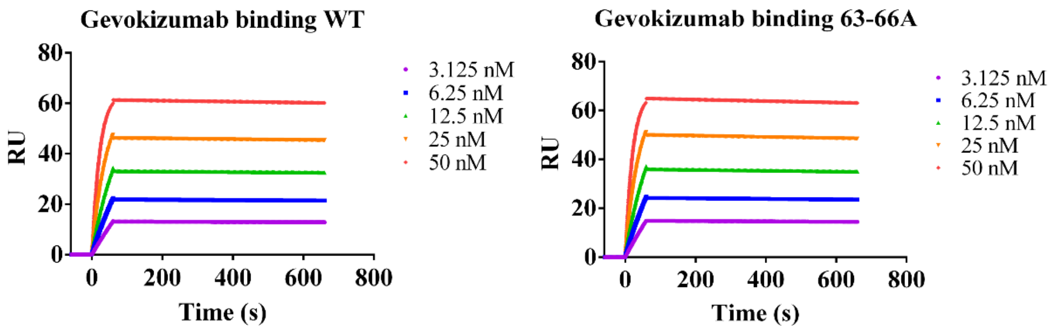

**ka (1/Ms):** 9.238E+5	**ka (1/Ms):** 1.100E+6
**kd(1/s):** 3.205E-5	**kd(1/s):** 4.923E-5
**KD(M):** 3.469E-11	**KD(M):** 4.477E-11

Figure S4. The affinity of Gevokizumab binding to WT hIL-1β and 63-66A, measured by SPR. Gevokizumab exhibited similar KD values to WT (34.7 pM) and hIL-1β63-66A (44.8 pM).

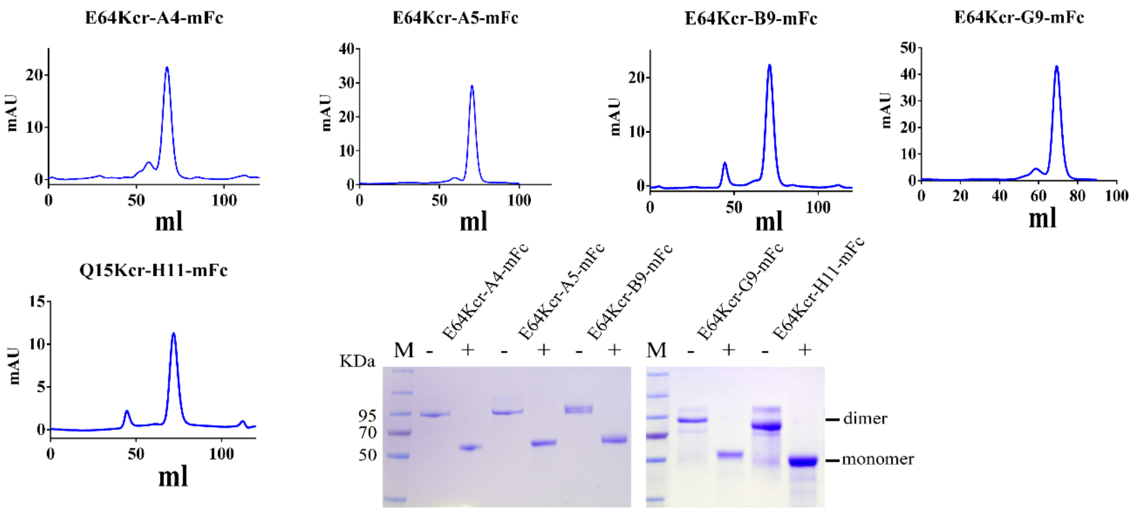

Figure S5. Protein A purified antibodies were analyzed by SEC (Superdex200) and SDS-PAGE.

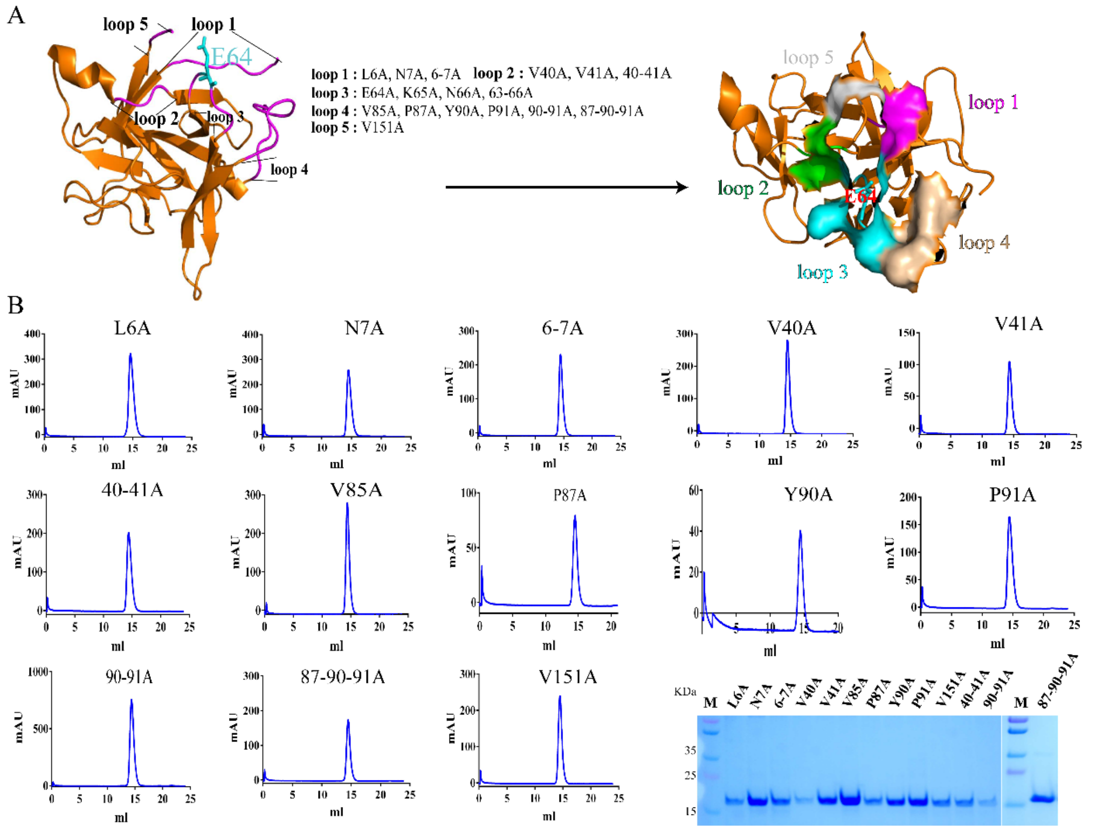

Figure S6. Ala scan mutation on target epitope (around E64 residue). (**A**)There are five flexible regions (loop1-5) near the target epitope. 17 Single-Ala mutations and multi-Ala mutations were chosen on each loop and generated. The left displayed the cartoon structure of hIL-1β, the right is the epitope surface of these 5 loops around E64 residue. (**B**) The purity of Ala mutants was analyzed by SEC and SDS-PAGE analysis. The purification of E64A, K65A, N66A, and 63-66A mutants can be found in our previous published paper (*20*).

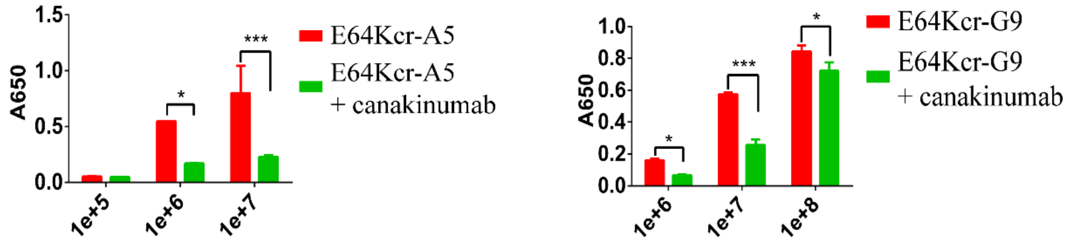

Figure S7. Verification of binding epitope of phage E64Kcr-A5 and E64Kcr-G9 by competition phage ELISA. Series diluted phage E64Kcr-A5 or E64Kcr-G9 were incubated with WT hIL-1β in the presence or absence of 300 nM canakinumab, and analyzed by phage ELISA.

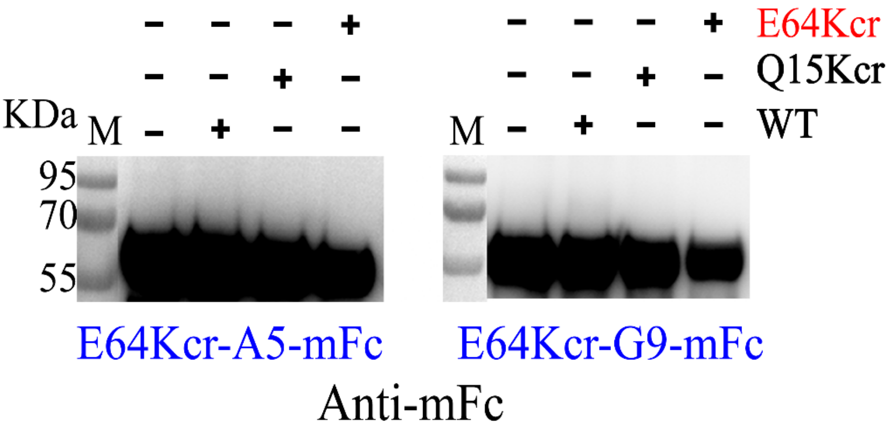

Figure S8. E64Kcr-A5-mFc and E64Kcr-G9-mFc do not crosslink with E64Kcr. 8 μM antigens were incubated with 4 μM E64Kcr-A5-mFc or E64Kcr-G9-mFc at pH 8.8 and 37°C for 2 days (48 hours), respectively. Anti-mFc antibody was used to detect whether there are crosslinked products.

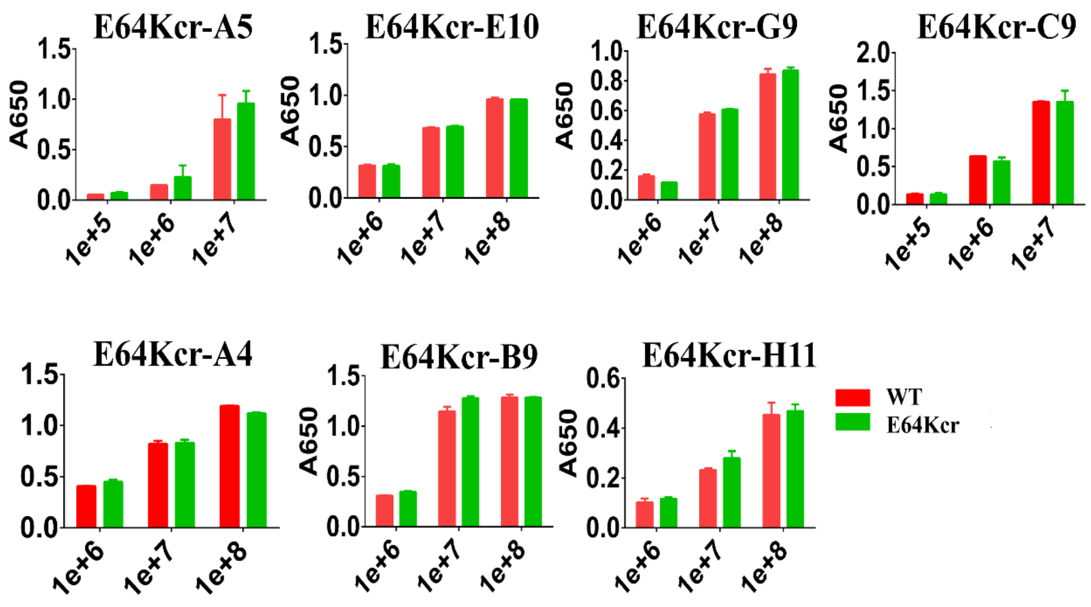

Figure S9. All selected phage hits showed similar affinity between WT hIL-1β and E64Kcr.

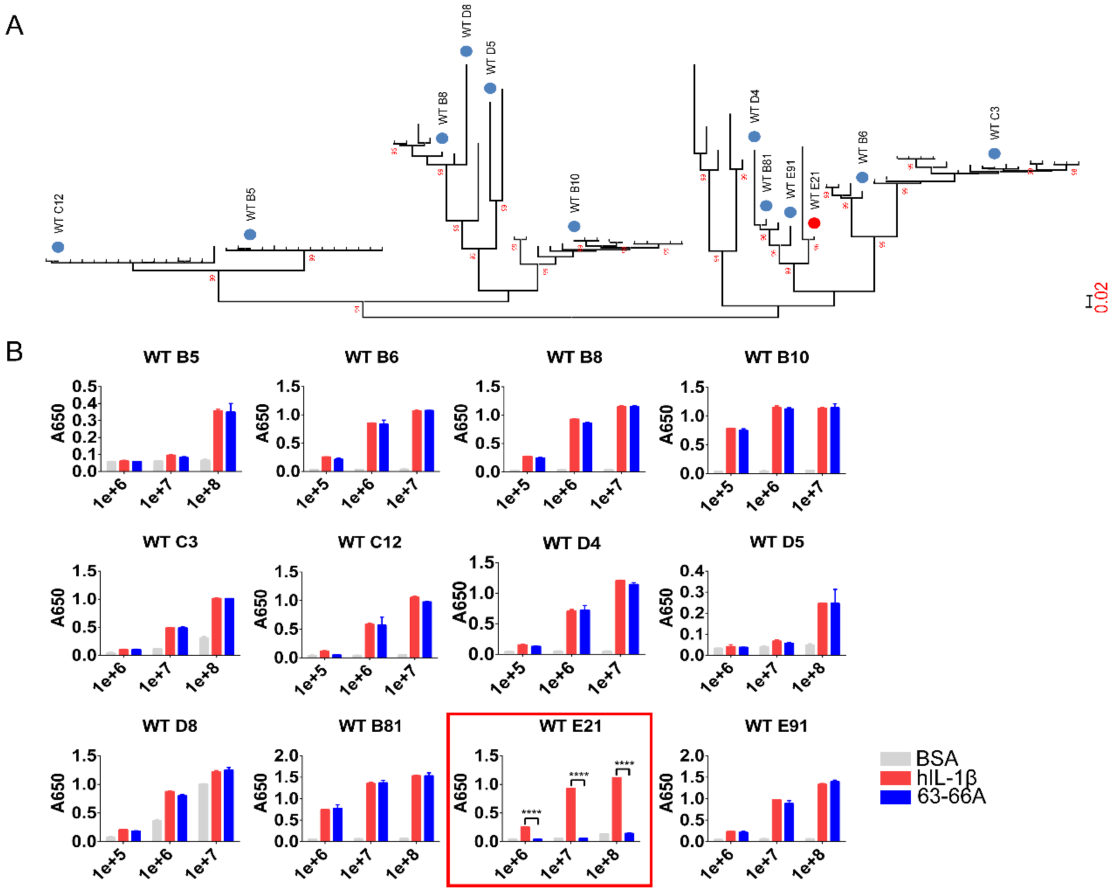

Figure S10. Epitope profiling of randomly selected monoclonal phages from WT hIL-1β immunization phage library. (**A**) After two rounds of conventional panning against WT hIL-1β, 96 clones were randomly picked for sanger sequencing. 87 clones containing full-length mouse scFv were analyzed by phylogenetic tree based on their amino acid sequence. The solid circle represents the clone selected for epitope profiling. The red dots are hits bound to the hIL-1β E64 epitope, while the blue ones are not. (**B**) 12 clones were selected based on their homology (consistency < 98%) for binding epitope verification. These monoclonal phages were analyzed and compared for their binding affinities to hIL-1β63-66A and WT hIL-1β by phage ELISA in pfu-titration. The antibodies showed reduced binding affinity to hIL-1β63-66A compared with WT hIL-1β are highlighted by a red box. Each independent ELISA experiment was performed with three technical repeats, the mean value of which is presented as one data column.

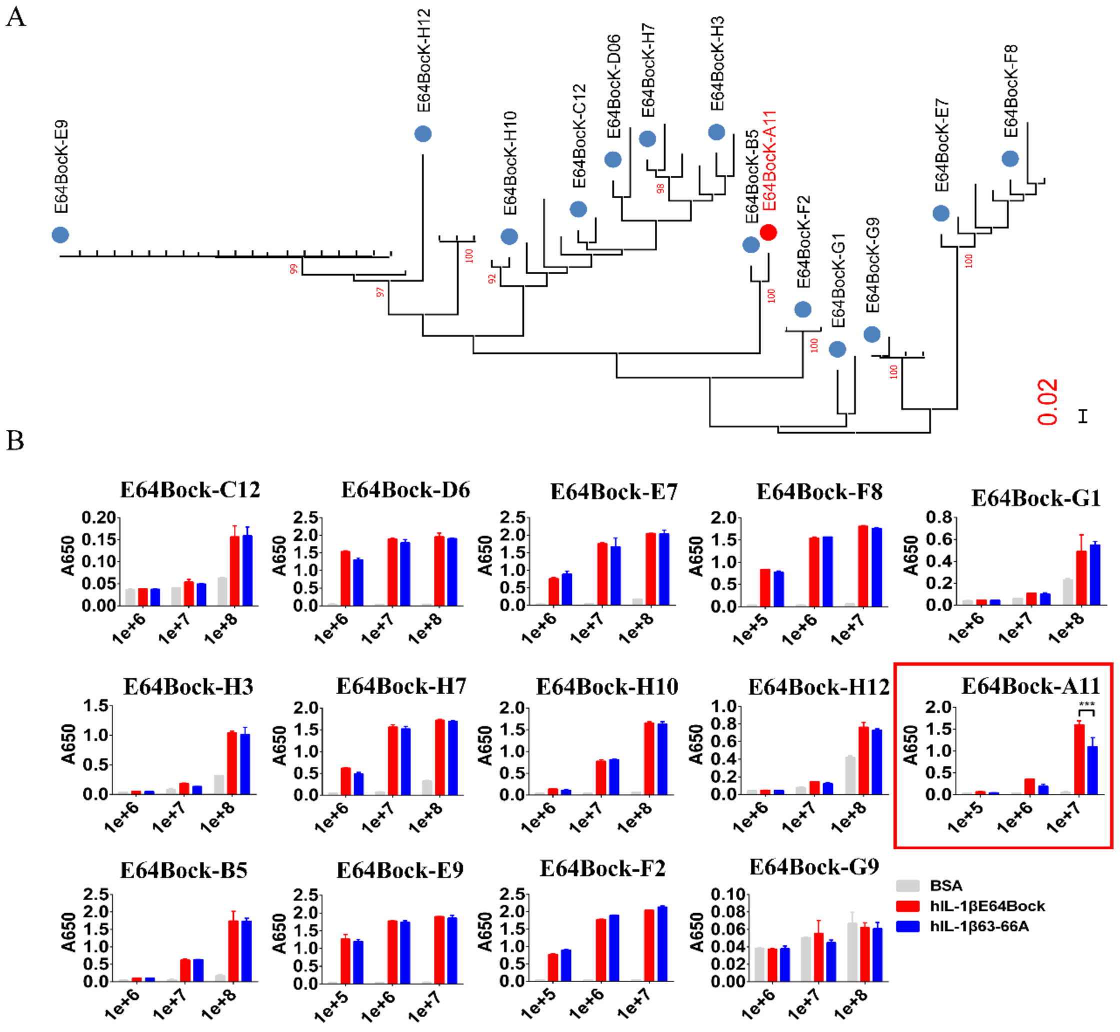

Figure S11. ELISA screening site-special antibodies binding to E64 epitope from E64BocK immune phage library. (**A**) After two rounds of traditional panning against E64BocK, 72 clones were randomly picked for sequencing. 58 clones containing full-length mouse scFv were analyzed by phylogenetic tree based on their amino acid sequence. The solid circle represents the clone selected for antigen binding epitope verification. The red ones are antibodies that bind to the hIL-1β E64 epitope, while the blue ones are not. (**B**) 14 clones were selected based on their homology (consistency < 98%) for binding epitope verification. These monoclonal phages were analyzed and compared for their binding affinities to hIL-1β63-66A and E64BocK by phage ELISA in pfu-titration. The antibodies showed reduced binding affinity to hIL-1β63-66A compared with E64BocK are highlighted by a red box. Each independent ELISA experiment was performed with three technical repeats, the mean value of which is presented as one data column.

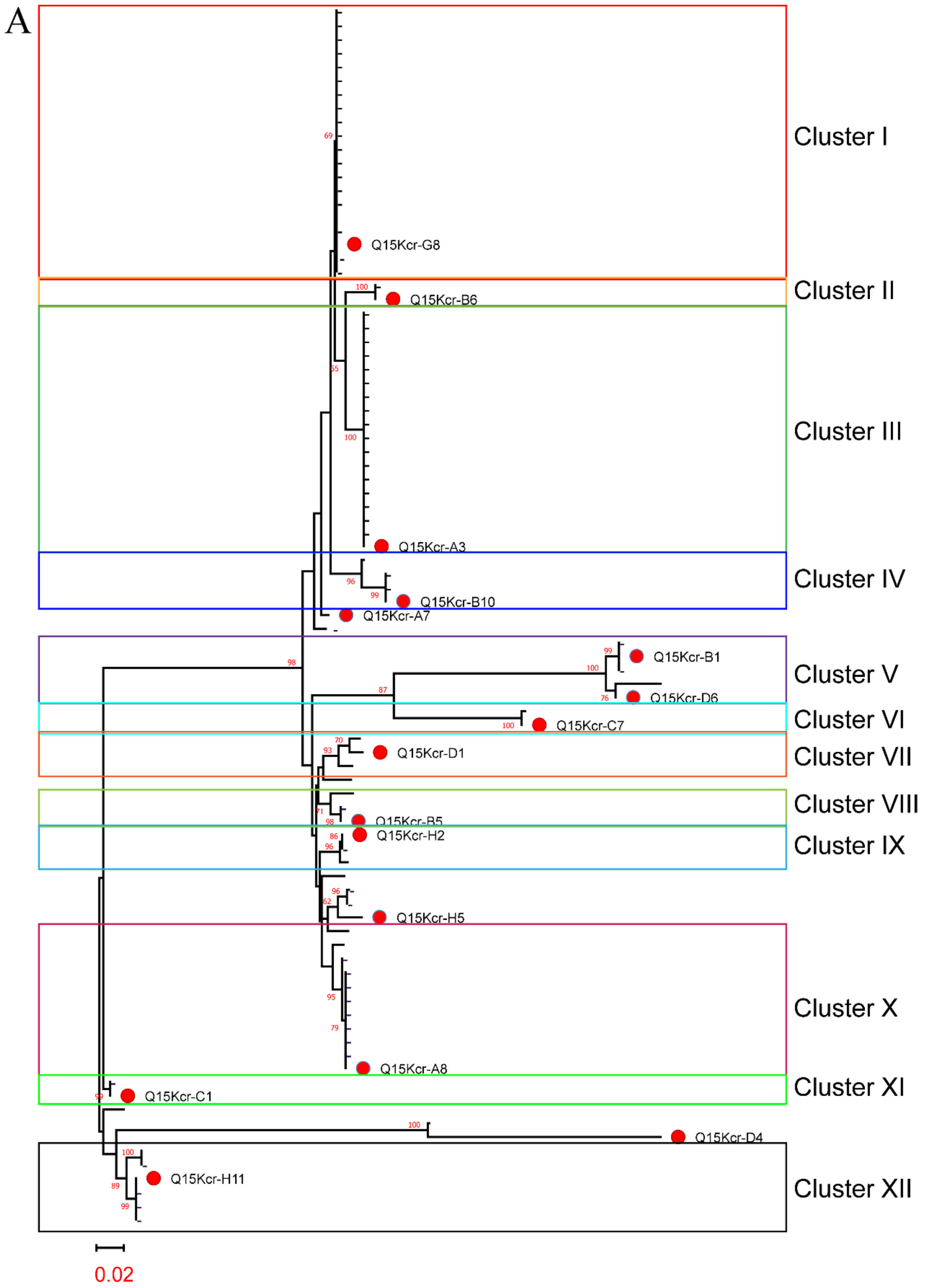

Figure S12. Sequence analysis of the output clones from panning Q15Kcr immunization phage library. 89 clones containing correct mouse scFv sequences were grouped into five clusters based on their amino acid sequence homology. The solid circle represents the clone selected for antigen binding epitope verification.

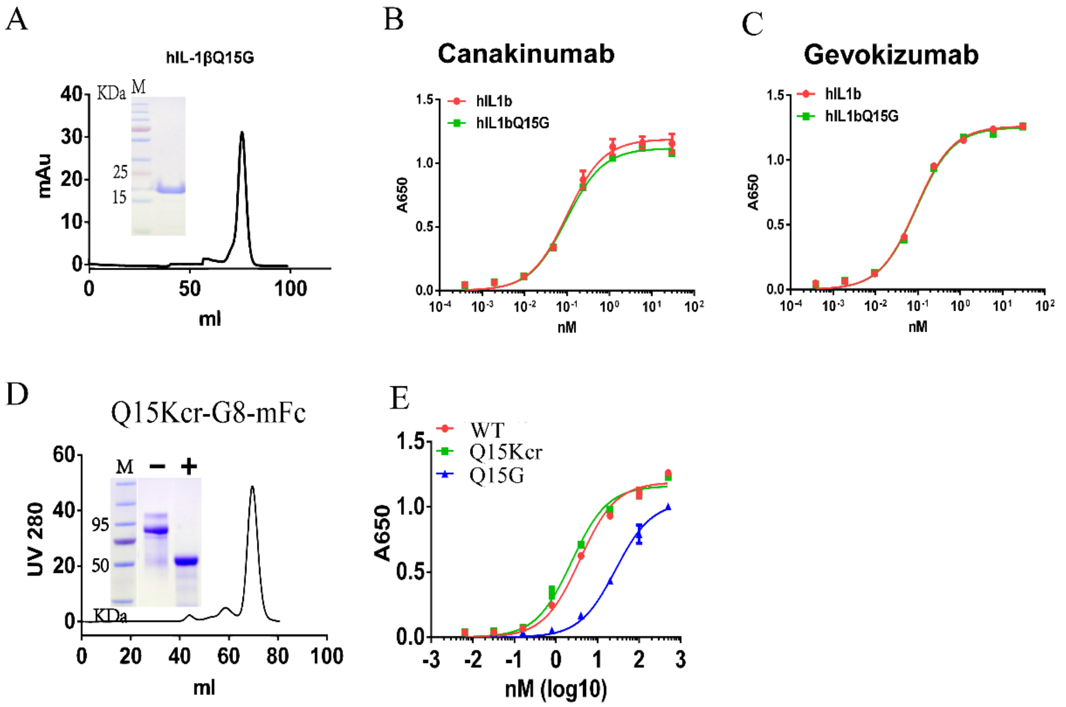

Figure S13. (A) Q15G was purified by Ni-NTA chromatography and analyzed by SEC and SDS-PAGE. **(B, C)** Both Canakinumab and Gevokizumab exhibited similar affinities between WT and Q15G, respectively, suggesting that this mutation of hIL-1β had no significant effect on its overall structure. **(D)** Q15Kcr-G8-mFc fusion antibody was purified by Protein A resin and analyzed by SEC and SDS-PAGE. **(E)** The affinity profile of Q15Kcr-G8-mFc binding to WT, Q15Kcr, and Q15G in a series of concentrations starting at 500 nM. The apparent K_d (± standard error of the mean, S.E.M.) of Q15Kcr-G8-mFc against WT, Q15Kcr, and Q15G were 3.8 ± 0.9 nM, 2.4 ± 0.6 nM, and 27.3 ± 6.3 nM, respectively.

A

B

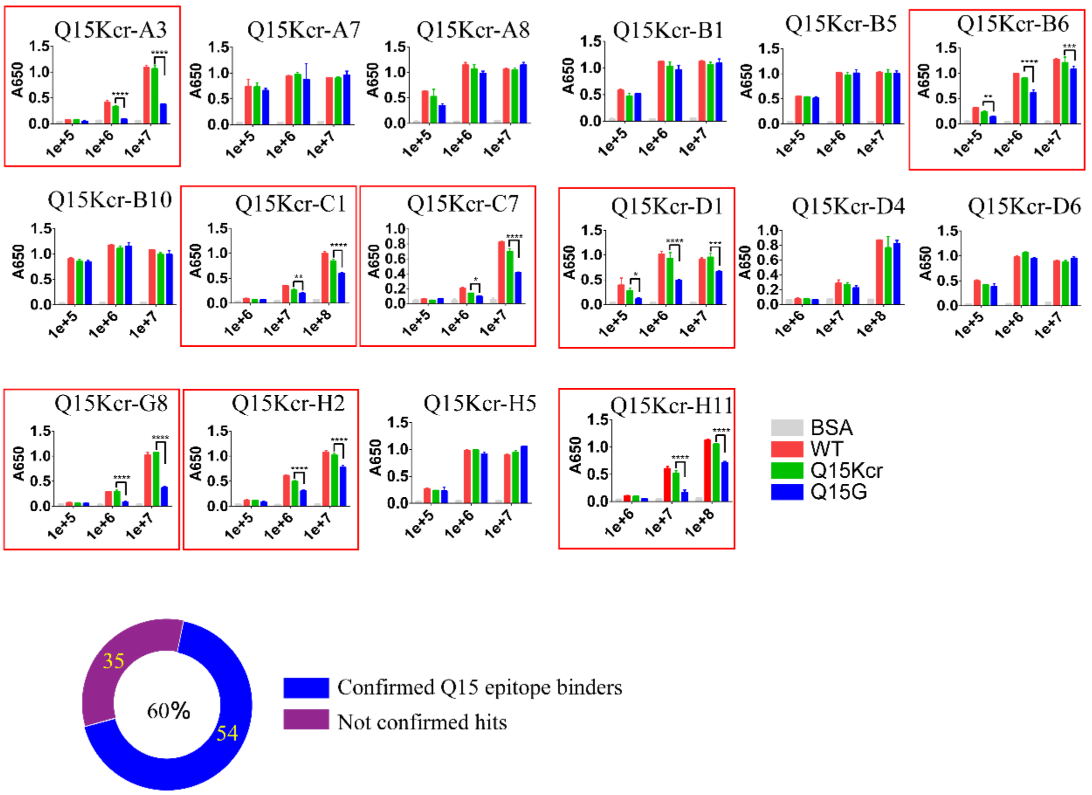

Figure S14. Kcr-induced epitope-specific antibodies response is independent of the epitope sequence. (A) identification of hits bound to hIL-1β Q15 epitope based on affinity difference between Q15Kcr and Q15G mutant by phage ELISA in pfu-titration. The X-axis is the number of phages (pfu). The antibodies showed reduced binding affinity to Q15G mutant compared with Q15Kcr are highlighted by a red box. Each independent ELISA experiment was performed with three technical repeats, the mean value of which is presented as one data column. *p<0.05; **p<0.01; ***p<0.001; ****p<0.0001. The detected 16 hits come from 12 different clusters, which cover almost all of the 89 analyzed hits. (B) The number and percentage are of antibodies binding to the Q15 epitope from the screened 89 hits based on affinity difference between Q15Kcr and Q15G mutant by phage ELISA.

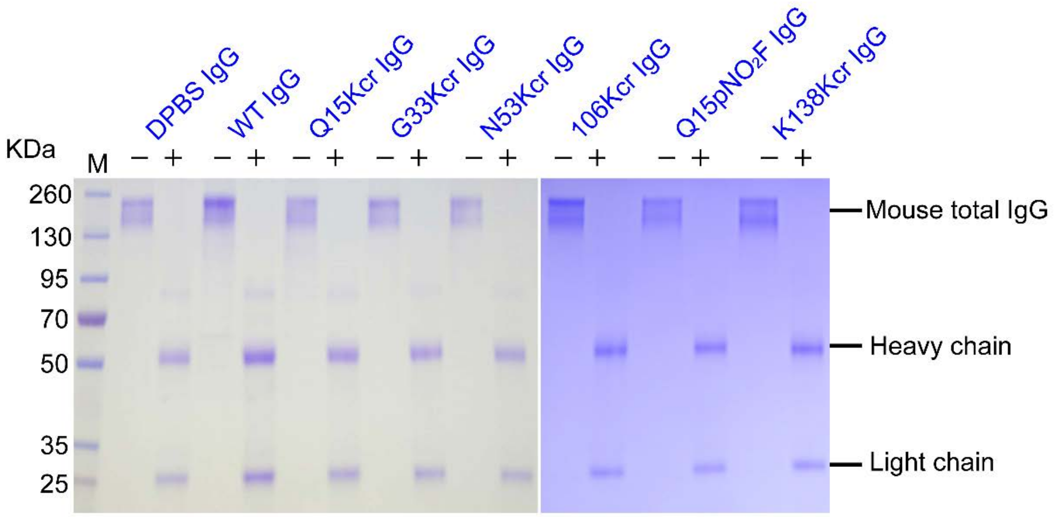

Figure S15. Total IgGs from each mouse immunization group were purified by protein A resin, and analyzed by SDS-PAGE. -, without DTT; +, with 10 mM DTT.

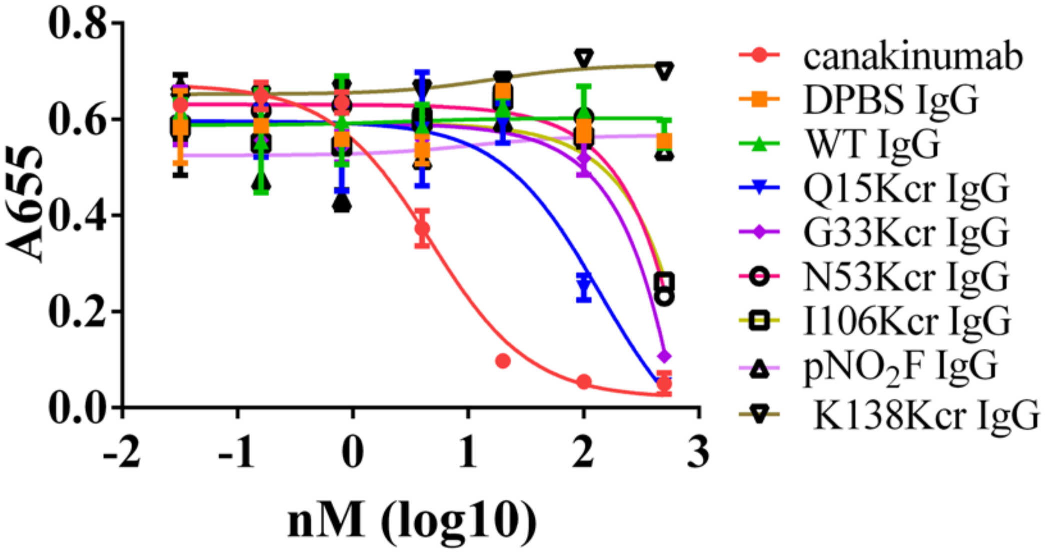

Figure S16. The dose-dependent inhibitory activities of total IgG from WT hIL-1β, Q15Kcr, G33Kcr, N53Kcr, I106Kcr, K138Kcr, and Q15pNO_2F immunized mice. Canakinumab was used as a positive control.

Table S1, ESI-MS results of ncAAs incorporated hIL-1β

Mutants	Calculated (Da)	MS (Da)
Q15Kcr	19048.76	19049.92
G33Kcr	19119.84	19121.91
N53Kcr	19062.79	19064.05
I106Kcr	19065.73	19065.09
K138Kcr	19119.79	19121.40
Q15AcrK	19036.48	19034.76
Q15pNO$_2$F	19115.75	19117.16
E64Kcr	18805.25	18806.64
E64AcrK	18791.25	18792.85
E64BocK	18838.28	18838.08

Table S2, the EC50 value of screened antibodies binding to hIL-1β and its mutants

	EC50 (nM, SE)				
Antigens	E64Kcr-A5-mFc	E64Kcr-G9-mFc	E64Kcr-A4-mFc	E64Kcr-H11-mFc	E64Kcr-B9-mFc
WT	0.034, 0.097	0.197, 0.057	0.121, 0.044	17.41, 0.015	0.040, 0.110
L6A	0.070, 0.08	0.555, 0.044	0.242, 0.054	19.45, 0.074	0.043, 0.113
N7A	0.038, 0.099	0.235, 0.042	0.150, 0.037	17.30, 0.043	0.054, 0.132
V40A	0.075, 0.100	0.383, 0.052	0.161, 0.063	18.30, 0.015	0.040, 0.107
V41A	1.147, 0.059	7.024, 0.046	0.713, 0.056	17.46, 0.026	0.050, 0.123
E64A	0.049, 0.121	0.193, 0.064	0.138, 0.051	15.50, 0.015	0.037, 0.101
K65A	0.065, 0.096	0.443, 0.027	0.219, 0.030	17.31, 0.018	0.049, 0.116
E64Kcr	0.057, 0.120	0.300, 0.053	0.154, 0.043	11.03, 0.032	0.032, 0.099
N66A	0.027, 0.096	0.153, 0.047	0.115, 0.034	10.06, 0.032	0.042, 0.107
V85A	0.085, 0.074	0.328, 0.025	0.193, 0.034	15.62, 0.019	0.043, 0.109
P87A	0.055, 0.119	0.303, 0.054	0.207, 0.035	28.96, 0.034	0.040, 0.104
Y90A	0.048, 0.099	0.303, 0.039	0.184, 0.069	24.83, 0.015	0.054, 0.110
P91A	0.066, 0.085	0.238, 0.047	0.188, 0.046	78.52, 0.036	0.051, 0.127
V151A	0.055, 0.108	0.208, 0.047	0.187, 0.037	9.04, 0.024	0.045, 0.111
6-7A	0.120, 0.094	1.035, 0.043	0.406, 0.052	13.25, 0.019	0.049, 0.126
40-41A	4.813, 0.083	29.90, 0.121	0.771, 0.040	16.37, 0.014	0.055, 0.135
90-91A	0.074, 0.078	0.235, 0.058	0.162, 0.042	66.31, 0.036	0.038, 0.099
87,90-91A	0.063, 0.109	0.344, 0.057	0.215, 0.030	67.58, 0.003	0.053, 0.131
63-66A	0.093, 0.083	0.469, 0.040	0.237, 0.051	41.26, 0.015	0.046, 0.114
